# Gene expression and metabolite profiling of gibberellin biosynthesis during induction of somatic embryogenesis in *Medicago truncatula* Gaertn

**DOI:** 10.1371/journal.pone.0182055

**Published:** 2017-07-27

**Authors:** Rafał Igielski, Ewa Kępczyńska

**Affiliations:** Department of Plant Biotechnology, University of Szczecin, Szczecin, Poland; United States Department of Agriculture, UNITED STATES

## Abstract

Gibberellins (GAs) are involved in the regulation of numerous developmental processes in plants including zygotic embryogenesis, but their biosynthesis and role during somatic embryogenesis (SE) is mostly unknown. In this study we show that during three week- long induction phase, when cells of leaf explants from non-embryogenic genotype (M9) and embryogenic variant (M9-10a) were forming the callus, all the bioactive gibberellins from non-13-hydroxylation (GA_4_, GA_7_) and 13-hydroxylation (GA_1_, GA_5_, GA_3_, GA_6_) pathways were present, but the contents of only a few of them differed between the tested lines. The GA_53_ and GA_19_ substrates synthesized by the 13-hydroxylation pathway accumulated specifically in the M9-10a line after the first week of induction; subsequently, among the bioactive gibberellins detected, only the content of GA_3_ increased and appeared to be connected with acquisition of embryogenic competence. We fully annotated 20 *Medicago truncatula* orthologous genes coding the enzymes which catalyze all the known reactions of gibberellin biosynthesis. Our results indicate that, within all the genes tested, expression of only three: *MtCPS*, *MtGA3ox1* and *MtGA3ox2*, was specific to embryogenic explants and reflected the changes observed in GA_53_, GA_19_ and GA_3_ contents. Moreover, by analyzing expression of *MtBBM*, SE marker gene, we confirmed the inhibitory effect of manipulation in GA_s_ metabolism, applying exogenous GA_3_, which not only impaired the production of somatic embryos, but also significantly decreased expression of this gene.

## Introduction

Somatic embryogenesis (SE) it is the process in which somatic (non-sexual) cells are induced to form bipolar embryos through numerous developmental steps similar to those occurring during zygotic embryogenesis. This process can occur in tissue and cell cultures of a great number of species as a result of a series of co-ordinated, highly organized cell divisions [1]. Competence to somatic embryogenesis is known to be highly correlated with the genotype, as exemplified by two *Medicago truncatula* embryogenic variants: 2HA and M9-10a [2, 3] which are considered as models for the study of SE in the species. Both lines were derived directly from non-embryogenic genotypes A17 and M9 respectively, which makes it possible to compare the process when SE is “switched on” or “switched off”. During somatic embryogenesis, some distinct developmental stages such as induction, differentiation and maturation can be conveniently distinguished. Each is regulated by specific physical and chemical factors among which plant hormones and plant growth regulators are considered to be the most critical. Among growth promoting substances, auxins and cytokinins are regarded as the major triggers of *in vitro* SE in angiosperm and gymnosperm plants [1, 4, 5]. However, our knowledge on participation of other plant growth regulators, especially phytohormones–and gibberellins (GAs) in particular–in regulation of SE induction and development of somatic embryos is still far from complete.

**Fig 1:**
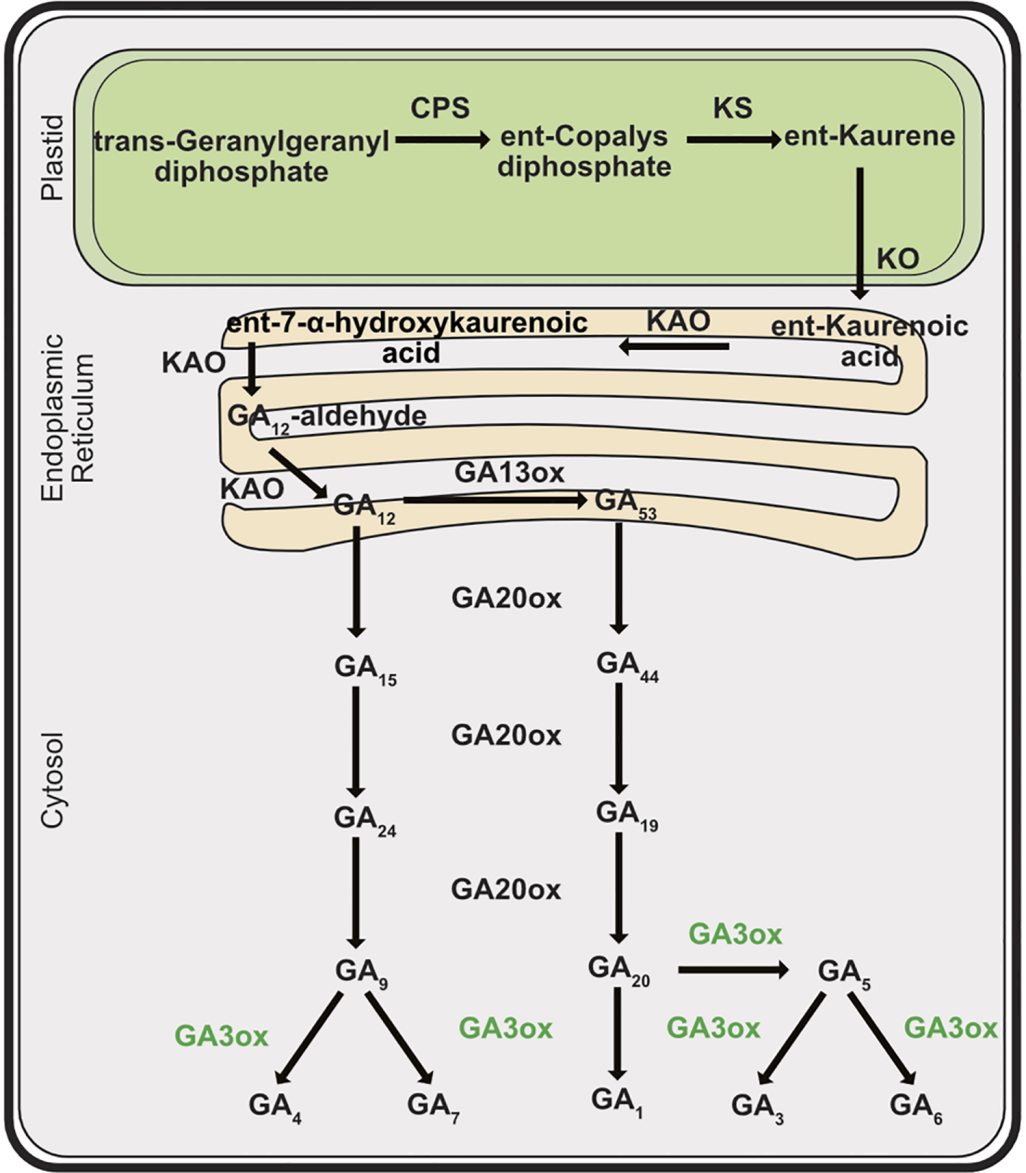
The gibberellins biosynthesis pathway in higher plants. The cellular localizations of metabolites are in plastids, endoplasmic reticulum and cytoplasm. Plant bioactive gibberellins are GA_4_, GA_7_, GA_1_, GA_3_, GA_5_ and GA_6_. Enzymes catalyzing subsequent reactions indicated in bold, abbreviations: *ent*-CDP, *ent*-copalyl diphosphate; (CPS), *ent*-copalyl diphosphate synthase; (KS), *ent*-kaurene synthase; (KO), *ent*-kaurene oxidase; (KAO), *ent*-kaurenoic acid oxidase; (GA13ox), Gibberellin 13-oxidase; (GA20ox), Gibberellin 20-oxidase; (GA3ox), Gibberellin 3-oxidase. Adapted from [7, 8].

Gibberellins, which belong to the tetracyclic diterpenoid class of hormones, comprise a group of over 136 natural plant constituents [6], but only some of them, e.g. GA_1_, GA_3_, GA_4_, GA_5_, GA_6_ and GA_7_ exhibit biological activity. Their biosynthesis is a multi-step process divided between plastids, reticulum and cytoplasm and effected by diverse enzyme families (Fig 1) [7, 8]. Biosynthesis of *ent*-kaurene is restricted to plastids and catalyzed successively by two enzymes: *ent*-copalyl diphosphate synthase (CPS) and *ent*-kaurene synthase (KS). The subsequent steps are associated with the endoplasmic reticulum and are catalyzed by cytochrome P450 monooxygenases: *ent*-kaurenoic acid oxidase (KAO) and gibberellin 13-oxidase (GA13ox) [9–11]. The gibberellin GA_12_, a product of KAO, is a substrate for cytoplasm-located gibberellin 20-oxidase (GA20ox) multi-family enzymes and follows a non-13-hydroxylation pathway leading to bioactive GA_4_ and GA_7_. On the other hand, GA_12_ may be 13-hydroxylated by GA13ox to GA_53_, an entry substrate for GA20ox in a pathway leading to bioactive gibberellins GA_1_ and GA_3_. In *Arabidopsis*, five *GA20ox* genes are known. The final step in which bioactive gibberellins are synthesized is carried out by gibberellin 3-oxidases (GA3ox), but the composition and levels are highly dependent on the species, tissue and process involved. The knowledge on gibberellins biosynthesis pathway is based mainly on data from *Arabidopsis* and *Oryza*, however knowledge regarding *Medicago* spp. remains residual.

Gibberellins play important roles in many aspects of plant growth and development, e.g. seed development and germination, somatic embryo germination and regeneration, stem elongation, leaf expansion and flower development [6, 7, 8, 12, 13]. Because somatic embryogenesis starts in most cases from leaf explants, so the primary status of gibberellin metabolism in leaf tissues should be considered with a particular attention. Expression of early (*CPS*, *KS*) and late (*GA20ox*, *GA3ox*) genes coding gibberellin biosynthesis enzymes was detected in leaves of *Arabidopsis* and *Pea* [14–16]. Active gibberellins were also found to be produced in mature leaves and transported through petioles and shoots, where they promote elongation, and further to the shoot apex where they regulate flower induction [17, 18]. At a later stage of somatic embryogenesis, differentiation resembles early stages of zygotic embryogenesis and it is then when embryos start to develop and differentiate. These morphogenetic events are also regulated by gibberellins; the lack of bioactive GA_s_ in gibberellin mutants caused seed abortion [19, 20]. Bioactive GA_1_ was found to increase during seed development; a distinct decrease was observed just before maturation and coincided with an increase in the ABA content [21–24]. The presence of gibberellin during this period may in part be an effect of suspensor activity which starts to develop at early embryogenesis from octant stage and then degenerates when embryos pass through heart stage [as reviewed 25, 26]. The complexity of the processes is a challenge when trying to translate the existing mechanisms to somatic embryogenesis.

Most of the information related to the role of gibberellins in SE comes from data obtained mainly from exogenous application of GA_3_ to different media at different stages of SE; the outcomes vary among species. On the one hand, exogenously applied gibberellins increased somatic embryo production in *in vitro* cultures of *Brassica oleracea* L. [27], *Cicer arietinum* L. [28], *Hardmickia binata* Roxb. [29], *Iris germanica* L. [30], *Medicago sativa* L. [31] and *Cocos nucifera* L. [32]. On the other hand, application of gibberellins was apparently successful not in all species: inhibitory effects of exogenous GA_s_ on somatic embryogenesis were observed in cultures of *Daucus carota* L. [33, 34], *Linum usitatissimum* L. [35], *Oncidium* [36], *Pelargonia* x *hortorum* Bailey [37], *Centaurium erythraea* Gillib. [38] and *Triticum aestivum* L. [39].

Some authors reported GA_s_ biosynthesis inhibitors such as ancymidole, paclobutrazol or uniconazole to enhance SE in plants from a wide group of families, including *Echinochla frumentaceae* [40], *Asparagus officinalis* L. [41], *Pelargonia* x *hortorum* Bailey [37], *Oncidium* [36], *Pinus taeda* L. [42] and *Centaurium erythraea* Gillib. [38]. On the other hand, the inhibitors mentioned above repressed the process in e.g. brussels sprout, cicer, iris, alfalfa and carrot [27, 28, 30, 31, 43]. Addition of these inhibitors during the induction phase resulted not only in a reduction of the number of embryos obtained on the differentiation medium but also in impairment of their development. Thus, the data obtained so far from experiments involving manipulation of the endogenous gibberellin level by exogenous application of both gibberellins and inhibitors of their biosynthesis point to participation of endogenous gibberellins in SE regulation.

Little information is available about the expression of genes encoding gibberellin metabolism enzymes and gibberellins content during SE. Two GA20-oxidases, three GA3-oxidases and two GA2-oxidases (enzymes of the last group are responsible for GA_s_ inactivation via GA 2-oxidation) was examined during *Dacus carrota* somatic embryogenesis [43]. Genes encoding GA20-oxidase and GA2-oxidase were being expressed continuously as SE progressed; on the other hand, both *GA3-oxidase* genes were up-regulated after SE induction. Likewise, Noma *et al*. [44] identified several GA_s_ (GA_1, 4, 7_) during carrot SE; these hormones were also found in undifferentiated cells from a non-embryogenic cell line. The very high levels of endogenous biologically active polar GA_s_ in carrot cultures were associated with the absence of embryogenic development. On the other hand, significantly higher levels of endogenous GA_s_ (GA_1,3,20_) were found in the embryogenic callus of maize, compared to those in the non-embryogenic callus [45]. Furthermore, Jimenez and Bangerth [45–47] and Jimenez *et al*. [48] did not find any difference in GA_s_ levels among cultures of grapevine, carrot and wheat showing different embryogenic characteristics. Thus, the few works concerning endogenous GA_s_ contents in cultures of non-embryogenic and embryogenic lines showed once again the ambiguity in data.

The activity of hormones may lead to signal transduction and transcriptional regulation carried out mainly by transcription factors. During the last two decades, numerous transcription factors were isolated and proven to be crucial components of the regulation network governing plant SE [as reviewed 49, 50]. Among them, AGAMOUS-Like15 (AGL15), LEAFY COTYLEDON2 (LEC2) and FUSCA3 (FUS3) contribute to regulation of gibberellin biosynthesis in *Arabidopsi*s *thaliana* by, respectively, stimulation of *GA2ox6* and inhibition of *GA3ox2* and *GA3ox1* genes, which in consequence leads to the reduction of the contents of bioactive gibberellins [51–55]. The most recent data on soybean SE confirmed the negative impact of exogenously applied GA_3_ on the process, leading to reduced accumulation of *AGAMOUS-Like18* (*AGL18)*, *ABA INSENSITIVE 3* (*ABI3)* and *FUS3* transcripts [56]. The authors referred to proposed a model of interactions between transcription factors and hormones in which a low gibberellin accumulation was positively correlated with production of somatic embryos.

In addition to the transcription factors described previously, another BABY BOOM (BBM) member of AP2/ERF superfamily is pivotal for induction of SE in various plants including *Nicotiana tabacum* [57], *Glycine max* [58], *Arabidopsis thaliana* [59] or *Theobroma cacao* [60], and is regarded as a suitable marker of the process. Only residual information refers to the significance of *BBM* for *Medicago truncatula* Jemalong, var. 2HA somatic embryogenesis where transcripts appeared after one week on induction medium [61]. There are no data regarding *BBM* regulation by gibberellins but a question remains if this may be part of known mechanisms accompanying regulation of somatic embryogenesis.

To the best of our knowledge, there are no detailed studies on biosynthesis of GAs, their role during the induction phase of plant SE, and their contribution to regulation of the expression of *BBM*, the SE marker gene. Therefore, the present study was conducted to: **i***—*investigate gibberellin biosynthesis by determining endogenous GAs contents and identification of the related genes their expression in the non-embryogenic genotype (M9) and embryogenic variant (M9-10a) of *Medicago truncatula* cv. Jemalong and **ii**—investigate effects of manipulating levels of endogenous gibberellins by adding to the induction medium GA_3_ and paclobutrazol (PBZ inhibitor of GA_s_ biosynthesis) on callus growth and subsequent somatic embryos production in connection with transcriptional activity of *MtBBM* SE marker gene. The results should provide new insights into the involvement of gibberellins in SE induction in plants, based on using the *Medicago truncatula* as a model to study somatic embryogenesis.

## Materials and methods

### Plant material

For mother plant production, we used seeds of two *Medicago truncatula* Gaertn.cv. Jemalong lines; non-embryogenic genotype (M9) and embryogenic variant (M9-10a), kindly provided by Professor Pedro Manuel Fevereiro, Instituto de Tecnologia Quimicae Biologica (ITQB), Portugal [3, 62]. Fresh seeds obtained from mother plants were ripened at 25°C for two months and then stored at– 20°C. Before sowing, the seeds were scarified using 96% H_2_SO_4_ for 8 min and then were rinsed five times with cold sterile water. Then, the seeds were placed in a sterile 15 cm Petri dishes (100 per plate) on Whatman filter paper moistened with water. The seeds were stratified in the dark at 4°C for two days and then were transferred to 20°C for 1 day. Seedlings with well-developed embryo radicle were placed in pots with sterile mixture of sand, soil, perlite and vermiculite (1:1:1:1). Plants were grown in a growth room at 24/22°C ±1°C day/night temperature, under a 16/8 h photoperiod of 120 μM m^-2^s^-1^ Green LED (Philips) for 2 months.

### Somatic embryogenesis protocol

**Fig 2:**
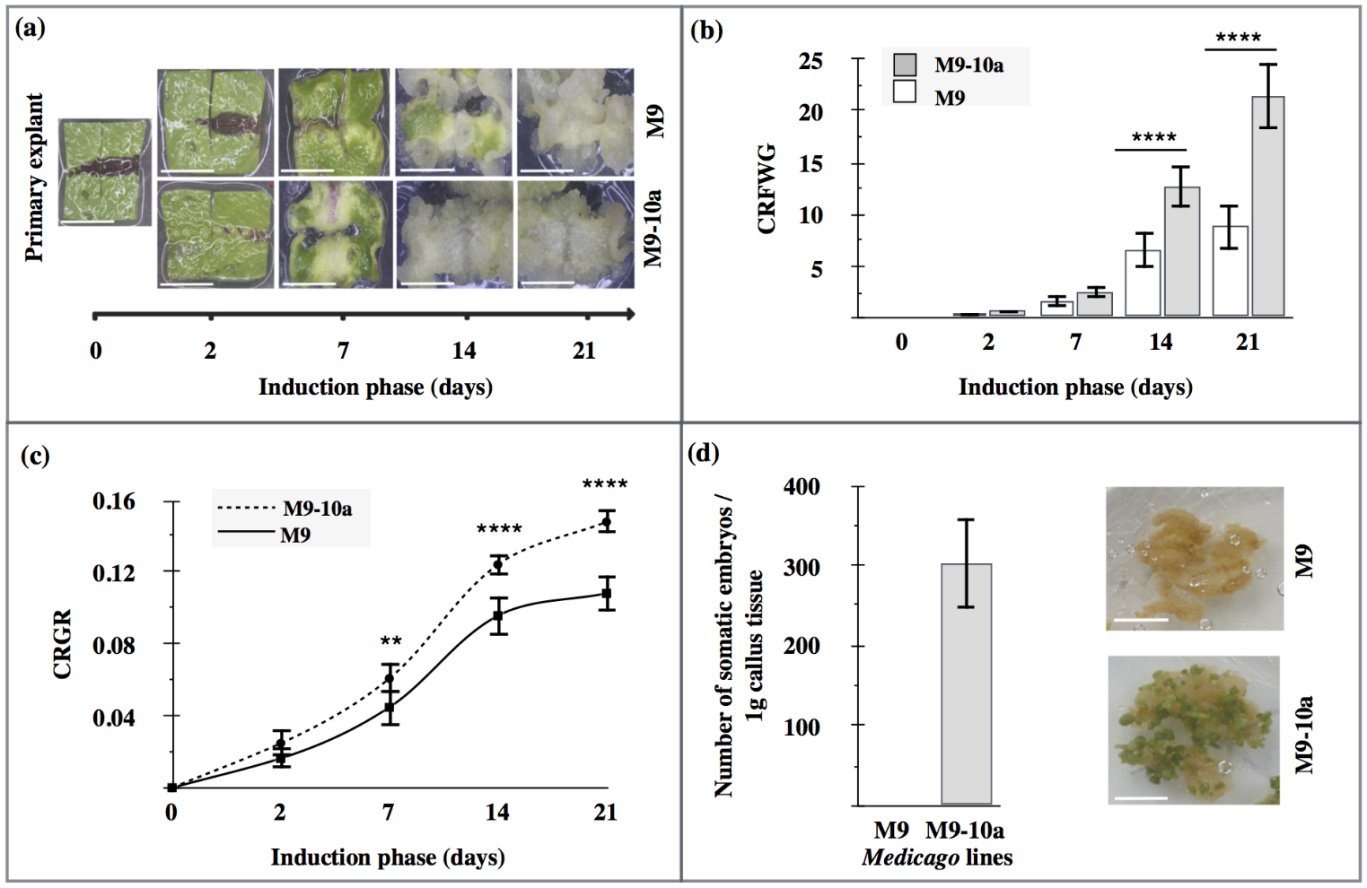
Development of callus and production of somatic embryos. (a) The leaf-to-callus transition on SH medium (21 days) from primary leaf explants of *Medicago truncatula* non-embryogenic genotype (M9) and variant with embryogenic phenotype (M9-10a). (b) Callus Relative Fresh Weight Growth (CRFWG) during induction phase of M9 and M9-10a explants. (c) Callus Relative Growth Rate (CRGR) during induction phase of M9 and M9-10a explants. (d) Somatic embryos production after three weeks of differentiation phase on MS differentiation medium. Statistical two-way ANOVA analysis with confidence interval 0.05 and Sidak post-hoc tests significance between groups indicated as two asterisks for P≤ 0.01 and four asterisks for P≤ 0.0001. Bars indicate +/- SD (n = 3). Scale bars 5 mm.

The somatic embryogenesis (SE) protocol consists of two steps which allow to separate the induction and the differentiation phases according to Araujo *et al*. [63] with some modification [13]. Fully expanded trifoliate leaves from the second and third node of a 2 month-old mother plant were excised and used as a source of initial explants. Leaves were surface-sterilized in 1% sodium hypochlorite for 5 min followed by washing three times in sterile water. The initial explants were prepared as squares of 1 cm x 1 cm size with one central cut perpendicular to the vascular bundles (Fig 2a). For induction callus formation, leaf explants were cultured for 21 days in a Petri dish (ø 55 mm) on SH medium [64] supplemented with 0.5 μM 2,4-D, 1 μM zeatin and 3% (w/v) sucrose. The medium was adjusted to pH 5.7 and solidified with 0.25% (w/v) gelrite. The cultures were maintained in a climate chamber in the dark and at 28°C. Subsequently, 21-old callus tissue was transferred onto the MS medium (differentiation medium) without hormones. The cultures were incubated in a growth room for 21 days at 24/22°C ±1°C day/night temperature, under a 16/8 h photoperiod with 70 μM·m^-2^s^-1^ GreenLED light intensity(Philips). To estimate the dynamics of callus growth, Callus Relative Fresh Weight Growth (CRFWG) and Callus Relative Growth Rate (CRGR) according to Huang *et al*. [65] were used. To analyze effects of exogenous gibberellin and paclobutrazol (PBZ) on SE, GA_3_ (0.5, 5, 10, 25, 50 μM) was added as a filter-sterilized aqueous solution to the induction SH medium; PBZ at the same concentrations was added prior to autoclaving. In experiment with the *BBM* gene expression to induction medium GA_3_ at 5 μM and PBZ at 10 μM concentrations were used. After 21 days of incubation on this medium the weight of cultures was determined. After transfer of the cultures and keeping them for next 21 days on MS differentiation medium, the somatic embryos were counted. There were at least 7 replicates (Petri dishes with trifoliate leaf explants on each) per treatment in each experiment. All the experiments described here were repeated at three times. Similar trends were obtained each time. The results of the experiments are presented as mean ± SD.

### Sample preparation for endogenous hormone quantification and qPCR analysis

Samples of both lines were collected at five time-points (day 0, 2, 7, 14 and 21) after placing the leaf explants in the induction medium; at last three biological replicates were obtained per a time point and each of the biological replicates consisted of seven individual trifoliate leaf explants cultivated independently on Petri dishes. For additional experiments, the same procedure was used but biological samples were collected at days 7 and 14. Samples were snap-frozen in liquid nitrogen and stored at −80°C until further analysis. All the analyses were conducted concurrently from the same samples.

### Determination of bioactive gibberellins and their precursors

To quantify the endogenous level of bioactive gibberellins and their precursors, samples were prepared as above and processed after storage at −80°C. The samples were analyzed for GA_s_ content according to Urbanová *et al*. [66] with some modifications. 20 mg of tissue culture material was homogenized in 2-ml Eppendorf tubes with 1ml of 80% acetonitrile containing 5% formic acid after addition of 15 GA internal standards ([^2^H_2_]GA_1_, [^2^H_2_]GA_3_, [^2^H_2_]GA_4_, [^2^H_2_]GA_5_, [^2^H_2_]GA_6_, [^2^H_2_]GA_7_, [^2^H_2_]GA_9_, [^2^H_2_]GA_15_, [^2^H_2_]GA_19_, [^2^H_2_]GA_20_, [^2^H_2_]GA_24_, [^2^H_2_]GA_44_, [^2^H_2_]GA_53_, [^2^H_2_]-GA_12_ and [^2^H_2_]-GA_12-ald_) (OlChemIm, Olomouc, Czech Republic) using an MM 301 vibration mill (www.retsch.de) at 27 Hz frequency for 3 min and 2-mm zirconium oxide beads were added to each tube to increase the extraction efficiency during homogenization. The tubes were then placed in a fridge (4°C) and extracted overnight with stirring using a benchtop laboratory rotator Stuart SB 3 at a frequency of 15 rpm (www.bibby-scientific.com). The homogenates were centrifuged at 20 000 rpm for 10 min at 4°C using a Beckman Avanti^™^ 30 centrifuge (Beckman Coulter Inc., Brea, CA). The supernatants were further purified using mixed mode anion exchange cartridges (www.waters.com) and analyzed in an ultra-high performance liquid chromatograph (Acquity UPLC^™^ System; Waters Milford, MA, USA) coupled to triple-stage quadrupole mass spectrometer (Xevo^®^ TQ MS, Waters MS Technologies, Manchester, UK) equipped with electrospray interface (ESI). GAs were detected using multiple-reaction monitoring mode (MRM) based on transition of the precursor ion [M–H]^−^ to the appropriate product ion. Data were acquired and processed by MassLynx^™^ 4.1 software (Waters Manchester, UK), and GA levels were calculated using standard isotope-dilution method [67].

### Identification and phylogeny of *Medicago truncatula* genes for components of the GA biosynthesis

To identify *Medicago truncatula* genes encoding the enzymes of the GA biosynthetic pathway, *Arabidopsis thaliana* protein sequences of known function from the TAIR data base, were used as queries to search the *Medicago truncatula* JCVI Mt 4.0v1 data base. The NGS transcription library of the M9-10a embryogenic line from all time-points during the SE induction phase were used for additional BLAST analysis conducted with the Geneious software (Biomatters Ltd). Protein BLAST were used to obtain protein sequences from the NBCI data base for *Brassica napus*, *Cicer arietinum*, *Glycine max*, *Oryza sativa*, *Pisum sativum*, *Phaseolus vulgaris*, and *Solanum lycopersicum*. For the sequence-based phylogeny, multiple sequence alignments of protein sequence were performed using the ClustalW. The phylogenetic analysis was conducted using the Geneious 6.1 software [68] with the Neighbor-Joining tree building method and Jukes-Cantor genetic distance model. The trees were resampled 1000 times using the bootstrap method and out-groups were used for rooting of phylogenetic trees. The candidate *Medicago truncatula* genes are summarized in S1 Table.

### RNA isolation and cDNA synthesis

Samples were collected as described above. Total RNA was isolated from 50 mg of frozen tissues in 1 ml TRIzol Reagent (ZymoResearch) using Direct-zol^™^ RNA-MiniPrep Kit (ZymoResearch) according to the manufacturer’s instructions. DNA contamination was removed by using DNase I (ZymoResearch). RNA was eluted in 30 μl DNase\RNase Free-water. The purity and concentration of RNA was evaluated with BioSpec-nano (Shimadzu) and by electrophoresis in 2% agarose gel. First-strand cDNA of each sample was synthesized from 500 ng total RNA in a 20 μl reaction volume using the High-Capacity cDNA Reverse Transcription Kit (LifeTechnologies) according to the protocol and then used for quantitative PCR (qPCR).

### Quantitative real-time PCR

Gene-specific primers for quantitative PCR were designed using the PrimerExpress^®^ Software v3.0 (LifeTechnologies). All the sequences and parameters are given in S1 Table. Quantitative PCR (qPCR) was performed with the SYBR Select Master Mix (Applied Biosystems) using the STEP ONE Real-time PCR System (LifeTechnologies) following the manufacturer’s instructions. The 10 μl reaction mixture contained 5 μl SYBR Select Master Mix, 0.2 μl 10 mM primers, 1 μl cDNA template, and 3.8 μl DNase/RNase-free distilled water. The expression profile of selected genes in the M9 and M9-10a lines during the SE induction phase was obtained using 1:4 cDNA dilution. Analyses for both lines were run on separate plates. Additionally, the Inter-Plate Calibrator analysis was performed on each plate according to the GenEX user guide to compare profiles on one plot. To confirm the changes in transcription level at day 7 and 14, 1:3 cDNA dilution was used and the analyses for both lines were performed on a single plate. Three biological replicates of each time point in three technical replicates were conducted. The qPCR reaction conditions were as follows: initiation at 95°C for 2 min, followed by 40 cycles of amplification with 15 s at 95°C for denaturation and 1 min at 60°C for annealing. The final extension was performed at 60°C for 1 min. The dissociation curves were analyzed to check for gene-specific amplification; no non-specific products were detected. The reaction efficiency was 95–100%, as tested using a standard curve for each primer pair. One reference gene, *ACTIN2*, was used based on the existing bibliography and the previously conducted onsite geNorm and NormFinder evaluation within a group of 5 candidate reference genes. For each gene, the relative expression was calculated and shown as a fold-change using 2^-ΔΔCt^ method [69], normalized to *ACT2* and relative to the lowest observed transcription (for day profiles) or relative to expression in the non-embryogenic line M9 (for particular day 7 and 14 comparisons). Computer analyses were performed using the GenEX software (MultiD Analyses AB, Sweden).

### Statistical analysis

All the experiments were carried out in biological triplicates. Changes in callus growth, embryos production and gibberellins content were analyzed using GraphPad Prism (GraphPad Software, USA). The results are expressed as mean ± SD. Statistical analyses were performed using the ANOVA with confidence level 95%. Particular post-hoc test for one-way and two-way ANOVA were Sidak test. Differences between the mean values were considered to be significant at p<0.01 or p<0.05. Change in gene expression among induction phase days were analyzed using the GenEX software (MultiD Analyses AB, Sweden). The results are expressed as mean ± SD. Statistical analyses were performed using the Student’s t-test and ANOVA with confidence level 95%. Particular post-hoc test for one-way and two-way ANOVA were Tukey-Kramer’s test. Differences between the mean values were considered to be significant at p<0.01 or p<0.05.

## Results

### Morphological changes in leaf explants from M9 and M9-10a *Medicago truncatula* lines in inductive and differentiating media

The callus development in leaf explants of *Medicago truncatula* cv. Jemalong non-embryogenic genotype (M9) and embryogenic variant (M9-10a) after 2, 7, 14 and 21 days during the induction phase and after 21 days in the differentiation medium was analyzed (Fig 2). Under *in vitro* conditions at 28°C in the dark, the explant tissues underwent successive morphological changes when on the SH induction medium. Although no changes visible to the naked eye could be observed after 2 days, explants of both lines started swelling and buckling after 7 days, a distinct primary callus appearing in place of cuttings (Fig 2a). These results correspond with callus growth as expressed as the callus relative fresh weight growth (CRFWG)[65]. After 7 days, CRFWG doubled in both lines (Fig 2b), but without any significant between-lines difference. During the second and third week of the induction phase, callus tissues developed substantially and by day 14 and 21 CRFWG increased, respectively, to 7 and 9 in M9 and to 13 and 22 in M9-10a (Fig 2b). However, comparing to the non-embryogenic line, the embryogenic one produced remarkably higher amounts of callus with higher growth ratios (Fig 2b). On day 21 of induction, the M9 line callus structure was compact and granular, green-yellow in colour, while the embryogenic M9-10a line callus was also granular but looser and light-yellow in colour (Fig 2a). During the induction phase, the callus growth rate in the two lines tested (Fig 2c), expressed as the callus relative growth rate (CRGR) [65], showed a characteristic sigmoid-curve progression. It is possible to discern two distinct growth phases: the lag phase, limited to the first week of induction, and the fast growth phase, associated with the last two weeks of induction (Fig 2c). Three week-old explants with well-developed callus tissue were placed in a hormone-free MS differentiation medium; after 21 day-long cultivation under low light, embryos developed only on the surface of the embryogenic calluses (Fig 2d), 300 embryos (all developmental stages) per 1 gram of callus being recorded. In contrast, the M9 callus formed no embryos.

### Effects of GA_3_ and PBZ present during induction phase on callus growth and production of somatic embryos

**Fig 3:**
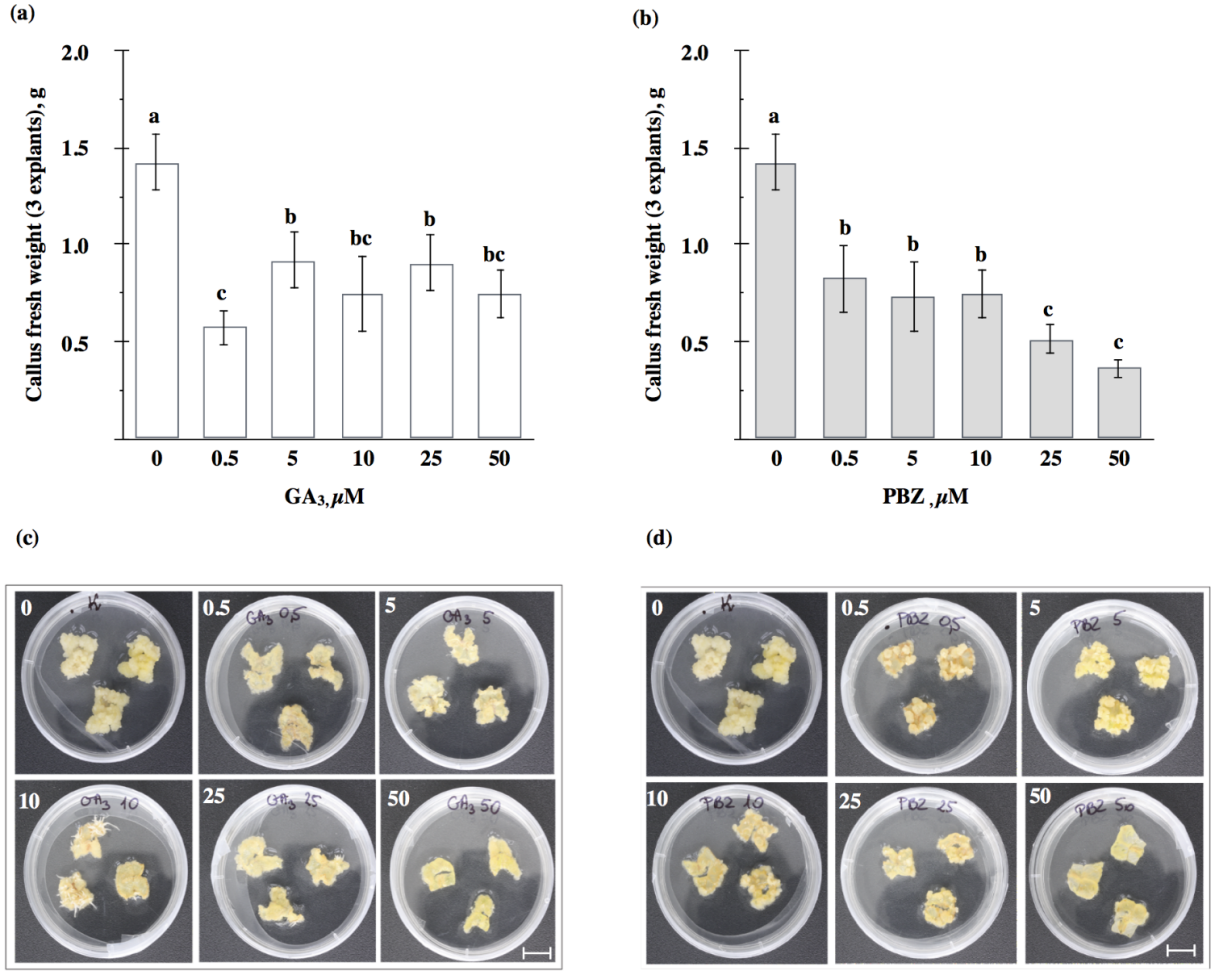
Development of callus after GA_3_ and PBZ application. Effects of GA_3_ (a, c) and PBZ (b, d) on callus growth on SH induction medium of *Medicago truncatula* embryogenic variant (M9-10a) after 21 days. One-way ANOVA with 0.05 confidence interval and Sidak post-hoc test; significance between groups indicated with letters. Bars represent +/- SD (n = 3). Bar scale 1cm.

**Fig 4:**
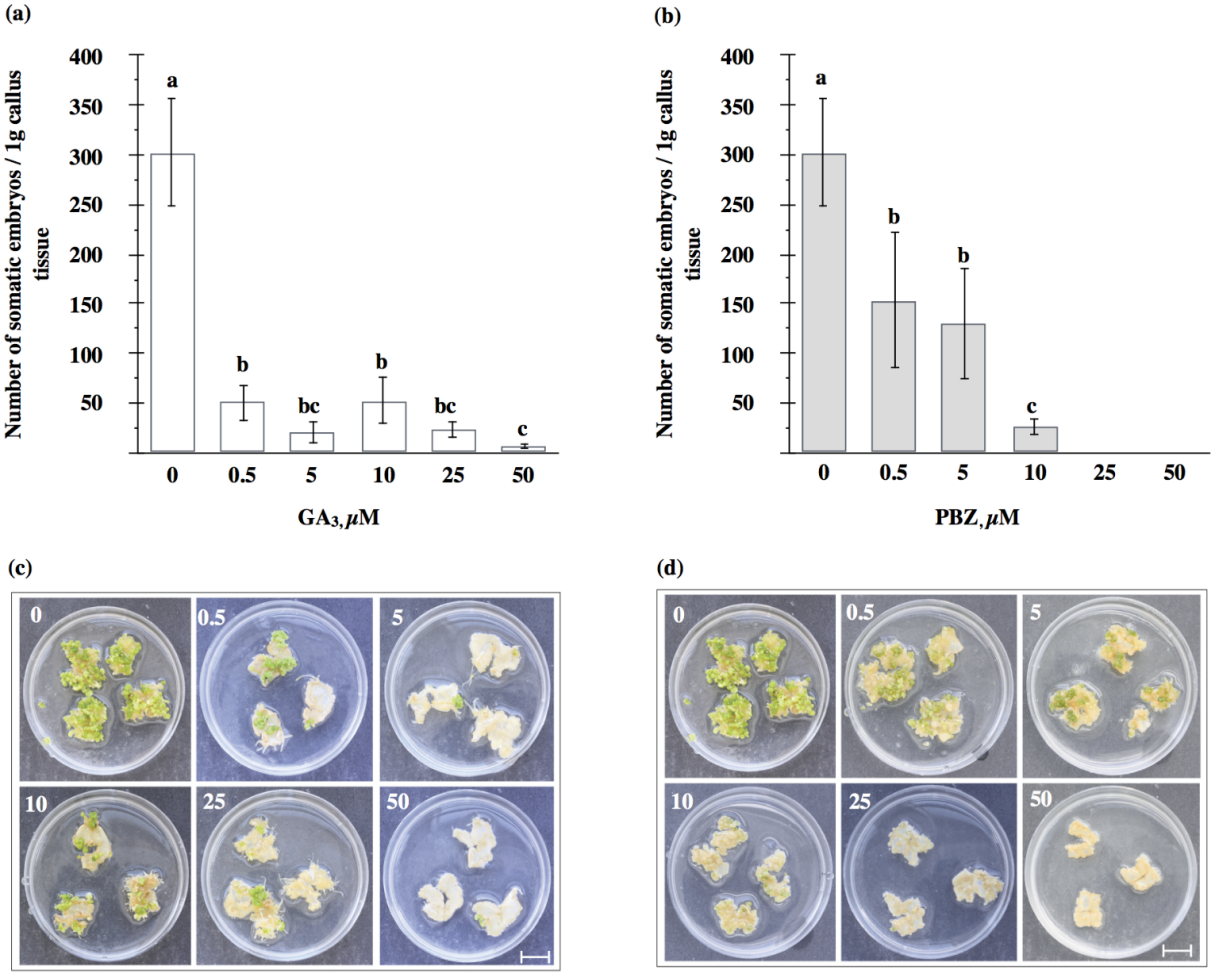
Production of somatic embryos after GA_3_ and PBZ application. Effects of GA_3_ (a, c) and PBZ (b, d) applied during induction phase on total somatic embryo production of *Medicago truncatula* embryogenic variant (M9-10a) after 21 days on MS differentiation medium. One-way ANOVA with 0.05 confidence interval and Sidak post-hoc test; significance between groups indicated with letters. Bars represent +/- SD (n = 3). Bar scale 1cm.

One possibility to examine effects of plant hormones on different morphogenetic processes, including SE, in plants has been to apply exogenously hormones or inhibitors of their biosynthesis to the medium. Addition of GA_3_ to the induction SH medium at all the concentrations tested (0.5, 5, 10, 25 and 50 μM) resulted in a significant inhibition of callus growth, from 36 to 60%, in M9-10a leaf explants, as measured after 21 days (Fig 3a). The maximum inhibition, to about 60% of the control, was observed at the 0.5 μM concentration. The callus developed only on the cut edges, was compact and yellow-orange in colour (Fig 3c). Interestingly, in the presence of GA_3_ at concentrations of 10 and 25 μM, roots appeared at the explant edges. Paclobutrazol (PBZ), a gibberellin biosynthesis inhibitor which inhibits *ent*-kaurene oxidase (KO) in plastids, caused inhibition of callus growth in all the concentrations tested (Fig 3c). Addition of PBZ at the highest concentration (50 μM) to the induction medium resulted in a nearly 75% inhibition of callus growth, compared to the control. The callus obtained was compact, orange-yellow in colour, and restricted to the cutting surface of the leaf blade (Fig 3d). Addition of GA_3_ to the induction medium inhibited the subsequent embryo production after 21 days on the differentiation MS medium deprived of any plant growth regulators (Fig 4a). A gradual decrease in the total somatic embryo production occurred with GA_3_ concentration increasing from 0.5 to 50 μM; inhibition after 21 days varied between 83 and 98%. There were visible differences between the control and the GA_3_ treatment in the location of somatic embryos within the callus tissue (Fig 4c). In the control explants, embryos tended to cover the whole surface of the callus, while somatic embryos in the treatment were preferentially produced in the form of spots. PBZ applied during the induction phase, too, exerted an inhibitory effect on the subsequent embryo production in the differentiation medium (Fig 4b). When applied to the SH induction medium at concentrations of 0.5, 5 and 10 μM, PBZ produced a 49, 57 and 98%, reduction, respectively, in the number of embryos in the differentiation MS medium. Moreover, when PBZ was applied at concentrations of 25 and 50 μM, a 100% inhibition relative to the control was observed. Embryos which appeared after the PBZ application tended to develop in the form of spots on the callus surface (Fig 4d).

### Changes in GA_s_ contents in tissues of *Medicago truncatula* during induction phase

**Fig 5:**
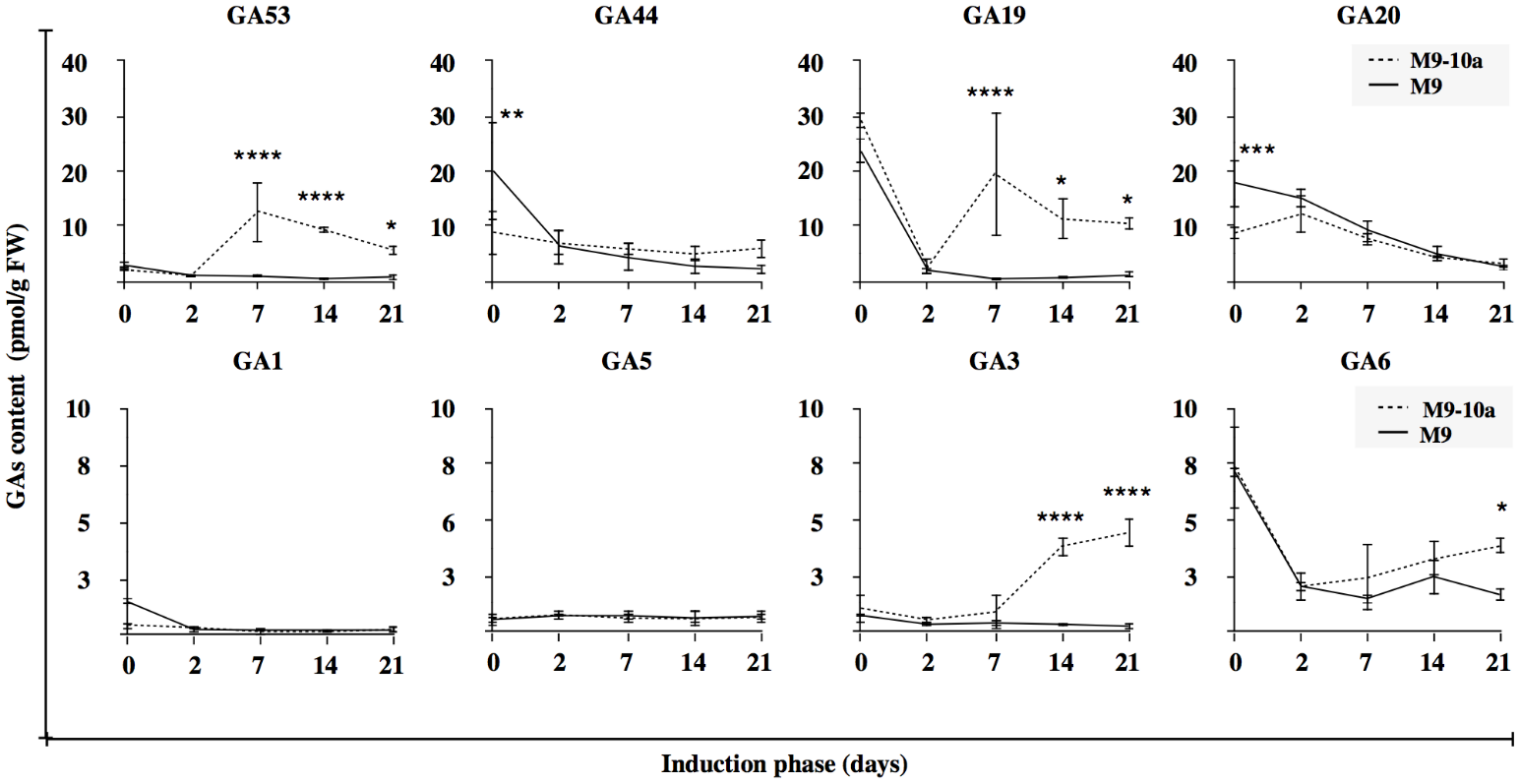
Changes in gibberellin content. Changes in endogenous levels of 13-hydroxy gibberellin metabolites during the induction phase in *Medicago truncatula* non-embryogenic genotype (M9) and variant with embryogenic phenotype (M9-10a). Tw-way ANOVA with 0.05 confidence interval and Sidak post-hoc test; significance between groups indicated with * for P≤ 0.05, *** for P≤ 0.001 and **** for P≤ 0.0001. Bars indicate +/- SD (n = 3).

The endogenous GA_s_ contents were analyzed in initial leaf explants and in tissues during the induction phase and callus development in the *Medicago truncatula* lines (M9, M9-10a) and assayed at day 0, 2, 7, 14, and 21. As illustrated diagrammatically in Fig 1, gibberellin metabolites are grouped into two pathways, whereby the production of bioactive gibberellins starts from GA_12_ and proceeds with or without C-13 hydroxylation, to form entry intermediates GA_53_ or GA_15_, respectively, as shown in Fig 5 and in S1 Fig. The biosynthesis of GA_12_-aldehyde and its metabolite GA_12_, predominant for GA_s_, was not detected in any of the samples tested. Primary leaf explants (day 0) contained all the gibberellins representing both pathways through GA_15_ to the bioactive GA_4_, GA_7_, (S1 Fig) and through GA_53_ to the bioactive GA_1_, GA_3_ (Fig 5). Non-bioactive gibberellins: GA_44_, GA_20_, GA_19_ occurred at the highest amounts, those of the first two were significantly (2-fold) higher in the M9 line, compared to the M9-10a line (Fig 5). GA_4_ was the most prominent gibberellin of all the bioactive gibberellins, and was equally abundant in both lines (6.65 and 8.88 pmol/g FW in M9 and M9-10a, respectively) (S1 Fig).

The 13-hydroxylation pathway (Fig 5) was significantly more active in the embryogenic line during the induction phase showing a particular increase in the amounts of GA_53_, GA_19_ and GA_3_. The amount of GA_53_ rapidly increased after the first week and then slightly decreased until the end of the induction phase to the amounts 6-, 4- and 3-fold higher, respectively, compared to the contents in the initial explants. At the same time, the contents of this gibberellin in the non-embryogenic line remained almost unchanged and were 14-, 19- and 7-fold lower, respectively, than those in the embryogenic variant. The amounts of GA_19_ in the M9-10a line on days 7, 14 and 21 were 48-, 18- and 10-fold higher, respectively than those in the M9 line, except for day 0 and 2 when the contents were similar in both lines. Changes in the content of these intermediates in the embryogenic callus were accompanied by an increase in the amount of the bioactive gibberellin GA_3_, 2-fold higher on day 7 and 10-fold higher on day 14, compared to the non-embryogenic callus. On the last day, the GA_3_ content in the M9-10a callus was up to 17-fold higher than that in the M9 callus. However, the content of another bioactive gibberellin, GA_1_ remained at the same low level in tissues of the M 9 and M9-10a lines during the entire course of induction phase, except for initial explants, and did not exceed 0.44 pmol/g FW. When changes in the non-13-hydroxylation pathway are compared (S1 Fig), differences between the embryogenic and the non-embryogenic line were observed in the tissue GA_15_ levels only. Throughout induction phase, the levels of GA_15_ in the non-embryogenic line showed a downward trend from 5.42 (day 0) to 1.33 pmol/g FW (day 21), greater changes being observed in the embryogenic line. After 2 and 7 days, the levels reached maximum (13.8 and 11.08 pmol/g FW, respectively), and after 14 and 21day, the GA_15_ amounts decreased to 5.08 and 4.18 pmol/g FW, respectively.

### Identification of genes coding enzymes of gibberellin biosynthesis leading to biologically active gibberellins

As mentioned earlier, plants possess numerous genes responsible for gibberellin metabolism. Phylogenetic analyses were performed to find and annotate *Medicago truncatula* genes, poorly identified so far. All the *Medicago truncatula* proteins used for alignment generation are summarized in S1 Table. The first enzyme in the pathway, copalyldiphosphate synthase (CPS), coded by a single gene in *Arabidopsis thaliana* (*At*) genome was confirmed in *Medicago truncatul*a (*Mt*) and its close relatives *Cicer arietinum* (*Ca*) and *Pisum sativum* (*Ps*) (S2a Fig). All of the *Fabaceae* CPS proteins were grouped in the same distinct clad. The BLAST analysis of *ent*-kaurene synthase (KS) revealed products of two genes in *Mt*, as opposed to just one each in *At*, *Ca*, *Phaseolus vulgaris* (*Pv*) and *Glycine max* (*Gm*). Interestingly, all of the *Fabaceae* proteins were grouped in the same clade and only the MtKS-like was notably out-grouped (S2a Fig). The phylogenetic tree generated for *ent*-kaurene oxidases (KO) (S2b Fig) has one protein for *At*, *Mt*, *Ps* and *Pv*, and all the *Fabaceae* proteins were grouped in one clade. Identification of ent-kaurenoic acid oxidase (KAO) revealed consistency in protein numbers (two of each) representing genes in the genomes of *At*, *Mt*, *Ca*, *Ps* and *Pv* (S2c Fig). Interestingly, another three *Mt* proteins which showed the highest percentage of similarity to *At* KAO counterparts appeared to be β-amyrin 11-oxidases and, together with other *Ca*, *Pv* and *Gm* proteins, formed a completely different clade. The gibberellin 13-oxidative enzymatic activity is specific to Gibberellin 13-oxidase (GA13ox) known also as Cytochrome P450—CYP714. The phylogenetic tree of CYP714 proteins (S2d Fig) showed *Mt*, similarly to *At*, to feature two possible CYP714A proteins of which any single one was grouped in different clades with other *Fabaceae* counterparts. Interestingly, two more *Mt* proteins: CYP714C1 and CYP714C2 were stacked into a separate clade with *Os* and other *Fabaceae*, but without significant similarity *At* proteins. The subsequent stages of gibberellin biosynthesis are catalyzed by cytoplasmic Gibberellin 20-oxidases (GA20ox), a multimember family which belongs to the oxidase family including also Gibberellin 3-oxidases (GA3ox), and both were separated into two distinct groups (S2e Fig). All GA20ox are located in two groups, and each of them consists of proteins representing all the species tested. The first group, in addition to *At* GA20ox5 assembled most of the *Mt* GA20ox proteins where these formed a *Fabaceae*-specific clade. The second group included all the remaining (four) *At* GA20ox and three *Mt* GA20ox. The phylogenetic trees of Gibberellin 3-oxidase enzymes which catalyze biosynthesis of bioactive gibberellins (i.e. GA_3_, GA_4_, GA_1_) revealed the presence of only two *Mt* proteins, compared to four *At* (S2f Fig). Moreover, all the proteins were divided into two distinct branches. All the *Fabaceae* proteins formed distinguishable clades, like in the former analysis. The results allow us to identify, in *Medicago truncatula*, 20 genes directly associated with bioactive gibberellin biosynthesis and 3 associated with biosynthesis of β-amyrin.

### Expression of genes involved in gibberellin biosynthesis during induction phase

The changes observed in the contents of gibberellins were coupled to molecular analysis of gene transcriptional activity. Because gibberellin biosynthesis is a multistep process, the results are divided into two parts for simplicity. The first part represents early steps, located in plastids and in the reticulum, the second one representing late biosynthesis steps taking place in the cytoplasm, which finally lead to bioactive gibberellins.

#### Early steps

**Fig 6:**
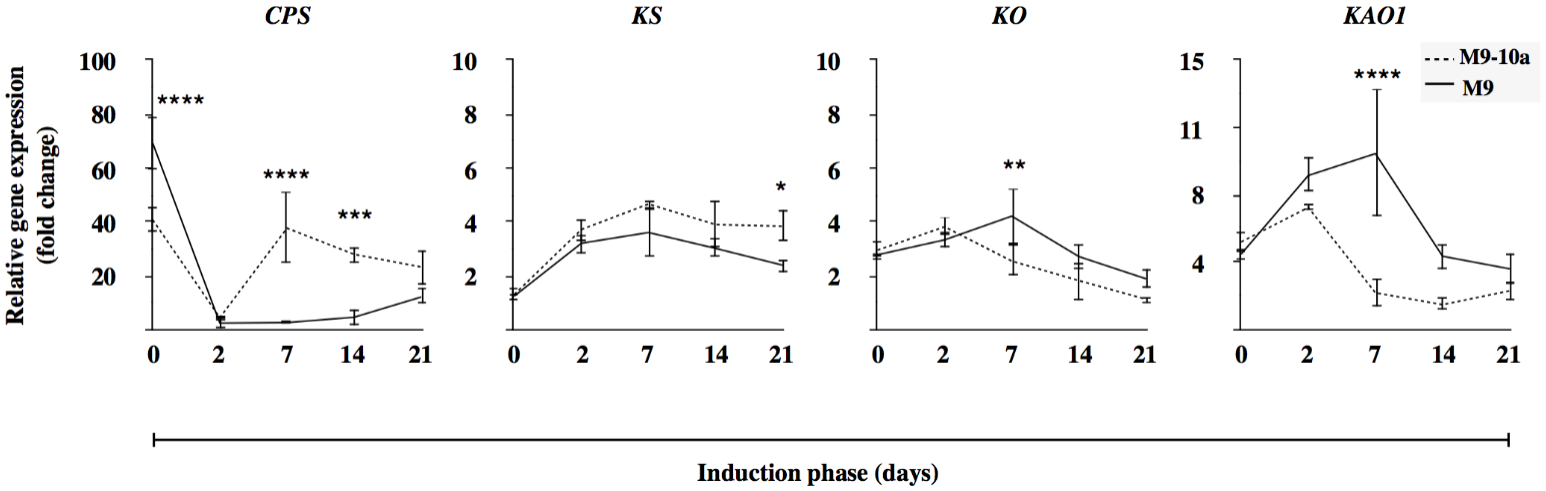
Relative gene expression of genes coding enzymes in early steps of gibberellin biosynthesis. *Medicago truncatula* genes: *CPS (ent-copalyl diphosphate synthase*), *KS (ent-kaurene synthase)*, *KO (ent-kaurene oxidase)*, *KAO (ent-kaurenoic acid oxidase)* expression profiles during induction phase (21 days) of *Medicago truncatula* non-embryogenic genotype (M9) and variant with embryogenic phenotype (M9-10a). Expression in both lines measured relative to the lowest observed expression set to 1. Two-way ANOVA with post-hoc Tukey-Kramer’s test with 0.05 confidence interval, significance between groups indicated with * for P≤ 0.05, ** for P≤ 0.01, *** for P≤ 0.001 and **** for P≤ 0.0001. Bars indicate +/- SD (n = 3).

**Fig 7:**
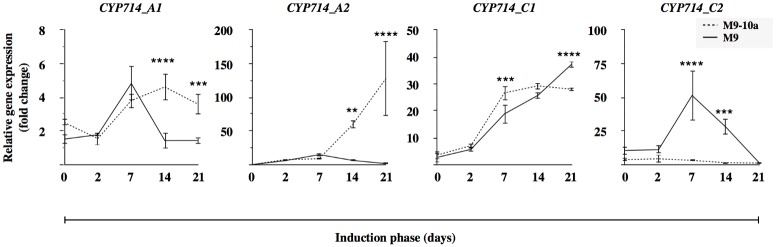
Relative gene expression of genes coding enzymes in intermediate steps of gibberellin biosynthesis. *Medicago truncatula CYP714* gene (*Cytochrome 450 family*, known also as *Gibberellin 13-oxidases*, *GA13ox)* expression profiles during induction phase (21 days) of *Medicago truncatula* non-embryogenic genotype (M9) and variant with embryogenic phenotype (M9-10a). Expression in both lines measured relative to lowest observed expression set to 1. Two-way ANOVA with post-hoc Tukey-Kramer’s test with 0.05 confidence interval, significance between groups indicated with ** for P≤ 0.01,*** for P≤ 0.001 and **** for P≤ 0.0001. Bars indicate +/- SD (n = 3).

In the subsequent steps, GA_s_ biosynthesis is carried out by CPS, KS, KO, KAO1 (Figs 1, 6 and S3 Fig) and cytochrome P450 proteins CYP714A1, CYP714A2, CYP714C1 and CYP714C2 which putatively have GA13-oxidase enzymatic activity (Fig 7). The relative gene expression was tested during three weeks of induction in non-embryogenic genotype (M9) and embryogenic variant (M9-10a) explants and presented as gene expression profiles relative to the lowest expression recorded within all the biological samples tested (Fig 6). In primary leaf explants, the expression of *CPS* in the M9 line exceeded that observed in the M9-10a line. After two days, the expression was rapidly reduced and reached the lowest value, similar in the two lines. Expression of *CPS* in the M9 line started to increase during the last two weeks only. On the other hand, the *CPS* gene expression in the M9-10a line was restored after one week to the initial level and remained at a similar level until the end of induction. A direct comparison of gene expression in both lines conducted on day 7 and 14 showed the expression in the embryogenic line to be 60- and 110-fold higher, respectively, compared to that in the non-embryogenic line (S3 Fig). The expression profile of the *KS* gene (Fig 6) was almost identical in both lines during the induction phase. The lowest expression was observed in primary explants; it then increased almost 4-fold during the first week of induction to remain at an almost unchanged level until the end. Analysis of the *KS* gene expression in both lines conducted on day 7 and 14 showed a 1.5-fold higher expression in the M9-10a line at the two time-points tested (S3 Fig). The *KO* expression profile was similar in both lines, the lowest expression being observed after the third week of induction and was the lowermost in the embryogenic variant (Fig 6), analysis on day 7 and 14 showed a slight lower expression in the non-embryogenic genotype (S3 Fig). Expression of the *KAO1* gene in the M9-10a line increased after two days to decrease and remain unchanged during the next two weeks of the induction phase. Expression in the non-embryogenic line gradually increased in the first week of induction and decreased on day 14 and 21 (Fig 6). Comparison of the *KAO1* expression in both lines on day 7 and 14 revealed that the expression in the M9-10a line was almost 10-fold lower on each of the days (S3 Fig). Expression of *CYP714A1* in the two lines was very similar, while at the first two time-points (day 0 and 2) it remained unchanged and was induced only after the first week (Fig 7). Since then, the expression differed between the lines: it decreased in the non-embryogenic line to the lowest observed level, whereas the embryogenic line explants showed the elevated expression until the end of induction. Only the expression analyzed on day 14 of induction differed between the two lines and was more than 3-fold higher in the embryogenic variant (S4 Fig). The expression profile of the *CYP714A2* gene (Fig 7) showed the gene to be up-regulated in both lines during the first week of induction; interestingly, transcripts were not detectable in primary explants. Expression during the two later weeks differed significantly between the lines tested and increased markedly in in the M9-10a line where it was up to 60- and 130-fold higher, compared to the lowest expression, observed on day 21 in the M9 line (Fig 7). Expression analyzed at the second week of the induction phase revealed a 12-fold higher expression in the M9-10a line (S4 Fig). The expression of the *CYP714C1* gene increased significantly between the second and the seventh day of the induction phase, in a similar manner in both lines (Fig 7). On the last day of the induction phase, expression observed in the M9 line exceeded that in the M9-10a line. Analysis of expression after the first and the second week of induction (S4 Fig) confirmed a nearly equal gene expression in the M9 and M9-10a lines. Expression of the *CYP714C2* gene measured in the embryogenic line remained almost unchanged and was close to the limit of detection (Fig 7). Expression observed in the M9 line in primary explants and on the second day was similar, but higher than that observed in the M9-10a line; subsequently, it markedly increased (on day 7 and 14), and eventually dropped to the level observed in the M9-10a line on the last day of induction.

#### Late steps

**Fig 8:**
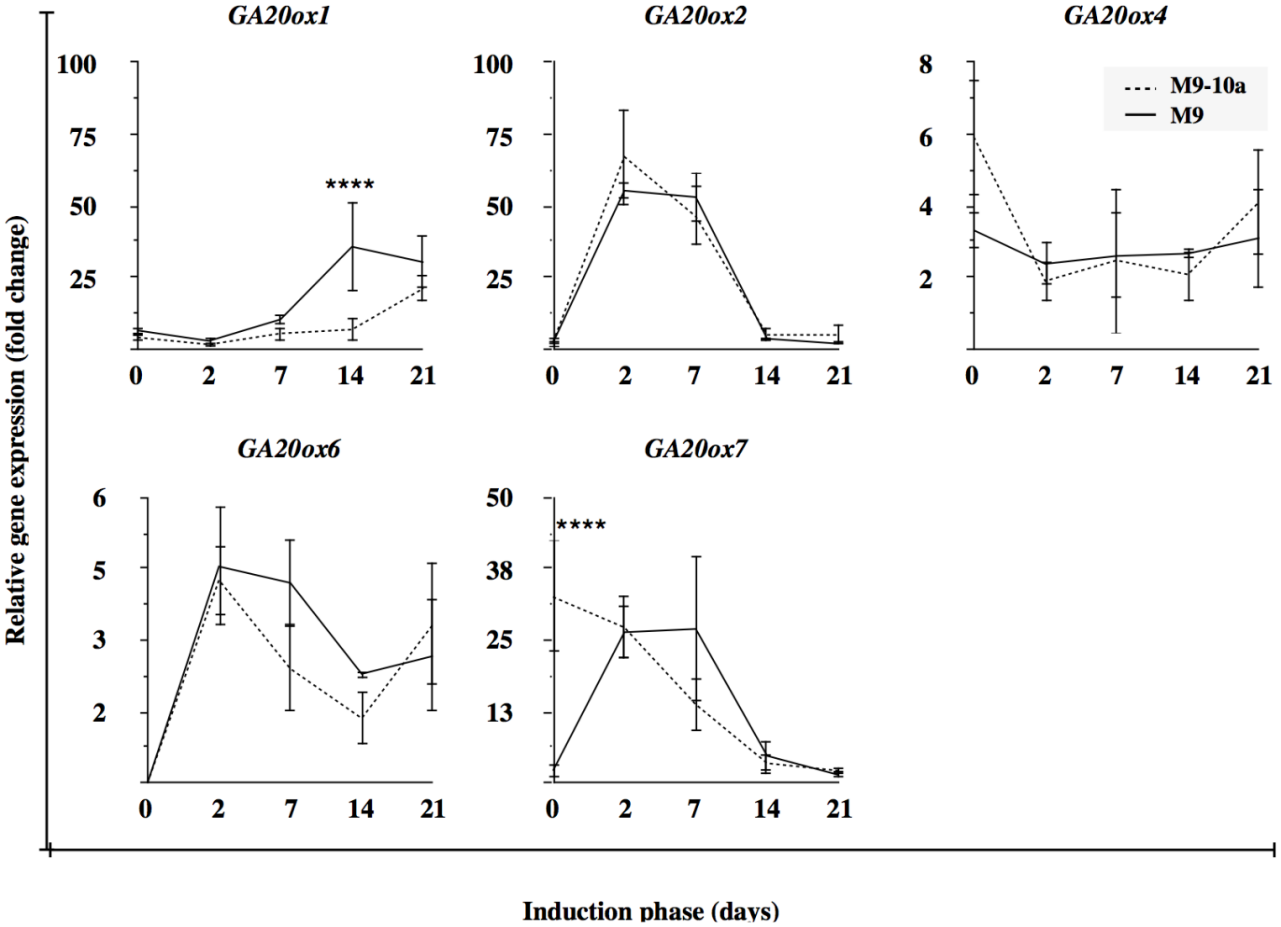
Relative gene expression of genes coding *GA20oxidases* in late steps of gibberellin biosynthesis. *Medicago truncatula GA20ox (Gibberellin 20-oxidase)* gene expression during induction phase (21 days) of *Medicago truncatula* non-embryogenic genotype (M9) and variant with embryogenic phenotype (M9-10a). Expression in both lines measured relative to lowest observed expression set to 1. Two-way ANOVA with post-hoc Tukey-Kramer’s test with 0.05 confidence interval, significance between groups indicated with **** for P≤ 0.0001. Bars indicate +/- SD (n = 3).

**Fig 9:**
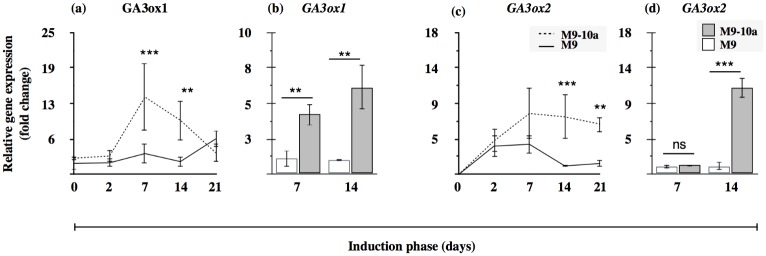
Relative gene expression of genes coding *GA3oxidases* in late steps of gibberellin biosynthesis. *Medicago truncatula GA3ox (Gibberellin 3-oxidases)* gene expression profiles (a and c) during induction phase (21 days) of *Medicago truncatula* non-embryogenic genotype (M9) and variant with embryogenic phenotype (M9-10a). Expression in both lines measured relative to lowest observed expression set to 1 and results are presented as a multiplication factor of change. Two-way ANOVA with post-hoc Tukey-Kramer’s test with 0.05 confidence interval. Expression of *GA3ox* genes (b and d) measured after first and second week of induction presented as a multiplication factor change in embryogenic variant (M9-10a) relative to non-embryogenic genotype (M9), set to 1. Statistical analyses were performed as two-tailed t-test with 0.05 confidence interval. Asterisks represent significance levels: **—P ≤ 0.001, ***—P ≤ 0.001 and ns for non-significant differences. Bars indicate +/- SD (n = 3).

In *Medicago truncatula* SE, the cytoplasmic enzymes Gibberellin 20-oxidases are represented by products of 5 active genes. The expression profile of *GA20ox1* during the first week (the first three time-points) was similar in both lines (Fig 8). Gene expression in the non-embryogenic line was significantly up-regulated during the last two weeks where its values were 35- and 30-fold higher relative to the lowest observed expression (on the second day in the M9-10a line). In the embryogenic line, expression started to increase between the second and the third week up to becoming 20-fold higher relative to the lowest value observed. On day 7 and 14, expression was 5- and 10-fold lower, respectively, in the M9-10a line, compared to that in the M9 one; these (and *GA20ox4* on day 14) were the only statistically significant differences for this type of analysis performed for all the *GA20ox* genes (S5 Fig). The expression profile of *GA20ox2* was almost identical during the whole induction period in both lines tested (Fig 8). The lowest expression was typical of primary explants, with values close to the limit of detection. Subsequently, expression markedly increased to reach the maximum values on day 2 and 7, expression being more than 50-fold higher, relative to that in primary explants. Finally, on day 14 and 21, the gene expression decreased almost to the primary level. Expression of *GA20ox4* in both lines, during the entire induction period was very low and remained almost unchanged, the differences having no statistical significance (Fig 8). The *GA20ox6* gene had a low overall expression in both lines. Transcripts of the gene were not detectable in any of the primary explants; subsequently, on the second day, expression in the two lines tested was 5-fold higher than that observed in the M9-10a line on day 14 which was the lowest (Fig 8). Expression in the M9 line remained almost unchanged on day 7, while it decreased in the M9-10a line to eventually become the lowest observed on day 14; at the same time, expression decreased also in the M9 line. The last gene tested, *GA20ox7*, was differentially expressed in primary explants of the M9-10a line with an about 30-fold higher expression relative to the M9 line (Fig 8). Expression of the gene in the M9 line increased on day 2 and 7 to reach the maximum and then to decrease. Its expression in the M9-10a line decreased systematically to the end of induction. The *M*. *truncatula* genome was found to feature two *GA3ox* genes coding putative enzymes responsible for biosynthesis of bioactive gibberellins. The first, *GA3ox1*, had the lowest expression in primary explants (day 0) of the M9 line. During the subsequent days, expression remained almost unchanged until day 21 when it increased 6-fold (Fig 9a). In the M9-10a line, expression of *GA3ox1* increased between day 2 and 7 to remain at an elevated level until day 14, and then finally decreased on the last day of induction to equal the expression of the M9 line. The relative *GA3ox1* expression on day 7 and 14 was 4- and 6-fold higher, respectively, in the M9-10a line than in the M9 one (Fig 9b). Transcripts of the second gene, *GA3ox2*, were undetectable in primary explants in any line. Subsequently, products of the gene were present in explants of both lines at day 2 (Fig 9c). Expression in the M9 line was unchanged also on day 7, to decrease thereafter to the lowest value on day 14 and 21. In contrast, expression in the M9-10a line increased after one week of induction and remained at a similar level until the end of induction, with a statistically significant difference at the last two time-points. Analysis of relative gene expression in the M9-10a line on day 7 revealed no difference with the M9 line, while on day 14, expression of the gene was 11-fold higher (Fig 9d).

### Expression of *BBM*, the SE marker gene, under GA_3_ and PBZ treatment

**Fig 10:**
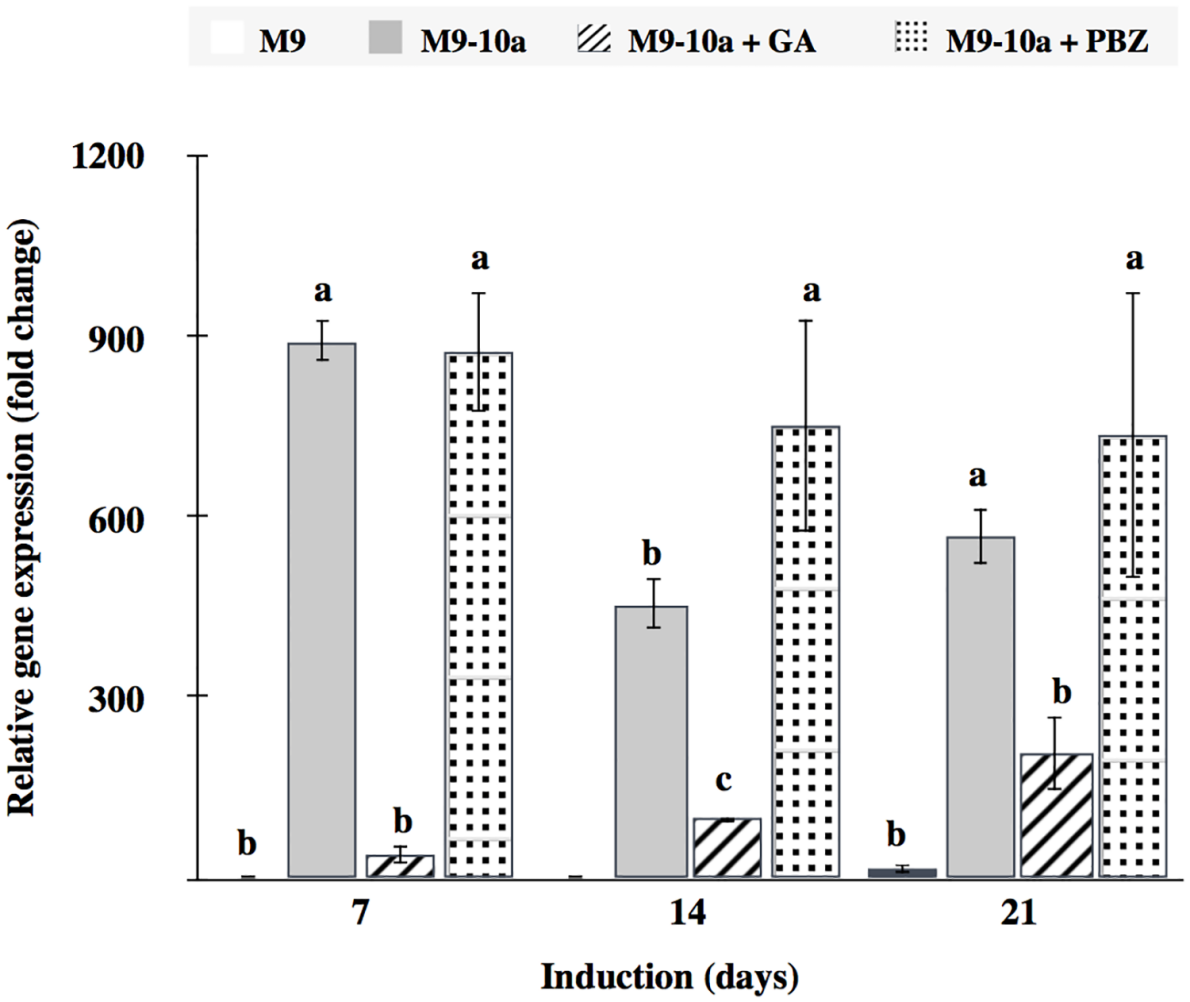
Relative gene expression of *MtBBM*. Effects of GA_3_ (5 μM) and PBZ (10 μM) on *Medicago truncatula BBM (BABY BOOM)* gene expression measured in the first, second and third week of induction, presented as a multiplication factor change in embryogenic variant (M9-10a) and embryogenic variant in the presence GA_3_ and PBZ (M9-10a + GA and M9-10a + PBZ) relative to the lowest observed expression in calluses of non-embryogenic genotype (M9) at day 7 set to 1. One-way ANOVA with 0.05 confidence interval and Tukey-Kramer’s post-hoc test; significance between groups indicated with letters. Bars represent +/- SD (n = 3).

Since transcription factor BABY BOOM (BBM) is known to be crucial for *Arabidopsis thaliana* SE, the *Medicago truncatula BBM* gene was used in this study to investigate the progress in acquisition of embryogenic competency in the plant. Also the effect of application of exogenous gibberellin GA_3_ (5 μM) and the gibberellin biosynthesis inhibitor, PBZ (10 μM), on expression of the gene was examined as well. The concentrations indicated were selected because, during the induction phase, their presence accompanied a significant, albeit incomplete, reduction in callus growth (Fig 3) and somatic embryo production (Fig 4). Transcripts of *BBM* were not detectable in any of the primary explants; neither were detectable on day 2 in any of the samples tested. Expression on day 7 in the M9 genotype was the lowest observed in all the samples and set as 1; then, expression in all other samples was presented relative to this level (Fig 10). Expression of *BBM* in the M9 line was non-detectable at the second week to become 14-fold higher on day 21 of the induction phase. The highest *BBM* expression on day 7 in the M9-10a line and after PBZ application was about 900-fold higher than expression in the M9 line. Expression of the gene in the embryogenic line decreased 450- and 570-fold on day14 and 21, respectively, relative to that in the M9 line on day 7. Application of PBZ in fact only slightly affect the *BBM* expression at 14 day of the induction phase. On the other hand, application of GA_3_ resulted in a significant reduction of the *BBM* expression which, on day 7, was just 40-fold higher than that in the non-embryogenic line the same day; subsequently, expression doubled in each subsequent week respectively to become 100- and 200-fold higher, respectively than the lowest expression in the M9 line on day 7.

## Discussion

Although much progress has been made in the past decades to understand the function of phytohormones such as auxins, cytokinins, and ethylene during SE initiation [reviewed 49, 70], knowledge on the role of gibberellins in this process remains highly incomplete [71–73]. This study focused on clarification of physiological and molecular basis in the function of gibberellins during the SE induction phase in *Medicago truncatula*, one of the model crop plants. Explanation of gibberellin metabolism during this phase could be based on developing a highly repetitive system, particularly with respect to light dependence of gibberellin biosynthesis [74]. Previous experiments established that *Medicago truncatula* SE may be induced similarly in light or in darkness [71, 75, 76, 77]. This study involved two *Medicago truncatula* lines with remarkably different phenotypes: the non-embryogenic (M9) and the embryogenic (M9-10a), the later one comes from somaclonal variation of M9 line. During the three-week long induction phase in darkness, the first week of induction ended with the residual callus being produced on cut edges of the embryogenic line explants; the changes visible during that period were slight only. Dedifferentiation and the formation of totipotent cells during the initial days is an effect of molecular mechanisms and activation of required transcription factors without any remarkable morphological changes [78, 79]. During the last two weeks of induction, a rapid growth of callus was observed, primarily in the embryogenic line. However, the difference in the callus structure between the two lines was not sufficient to determine the embryogenic potential (Fig 2a). Thus, it is not possible to determine when the formation of totipotent cells starts or ends, so according to Almeida *et al*. [77] it would be more appropriate to name this stage the “expression phase” rather than the “induction phase”. So, the three-week long induction phase in the embryogenic line (M9-10a) involves two distinct stages: dedifferentiation and expression. When calluses were transferred to a hormone-free medium, the somatic embryos started to develop, but on the embryogenic line explants only (Fig 2d).

### Gibberellin requirement for callus growth during induction phase and subsequent embryo production

As previously stated, there are contrasting reports about GAs involvement in SE, as evidenced by data produced by experiments employing application of exogenous GAs and their biosynthetic inhibitors, including paclobutrazol. This study demonstrated that GA_3_ applied even at low concentrations, 0.5 μM, during the induction phase caused inhibition of callus growth to 60% of the control (Fig 3a and 3c) and inhibition of the subsequent embryo production to 83% (Fig 4a). The results obtained are contrary to the outcome of our earlier studies; however, those studies were conducted in a different SE system, *Medicago sativa* L. cv. Rangelander, where GA_3_ at 5μM enhanced the number of somatic embryos to half of the control [31]. However, Hutchinson *et al*. [37] showed the addition of GA_3_ to the induction medium at a concentration of 1 μM to cause a 60–80% reduction in the number of embryos formed in *Pelargonium x hortorum* Bailey. In contrast, a positive effect of GA_3_ addition of to the induction medium on somatic embryo production has been reported for *Tylophora indica* (Burm.f.) Merrill (10 μM) [80] and *Cocos nucifera* L. (0.5 μM) [32]. This may suggest that the level of endogenous GAs may in some cases be sufficient, and in other not, for the proliferation of embryogenic callus and embryo development. Probably, in the *Medicago truncatula* M9-10a line, amounts of endogenous gibberellins were adequate for the progress of SE.

Paclobutrazol, a triazole inhibitor of ent-kaurene oxidase (KO) (Fig 1), when present in the SH induction medium, strongly inhibited the callus growth and subsequent production of somatic embryos in the *Medicago truncatula* embryogenic line up to 75% (Fig 3b and 3d) and 100% (Fig 4b and 4c), respectively, compared to the control. Those effects may indicate that endogenous GAs are required for both these processes, and reduction of their levels results in SE impairment. This was confirmed by results obtained previously in the *Medicago sativa* SE system [31]. However, there are also data showing a positive effect of paclobutrazol on SE [38, 42]. To summarize, the response of embryogenic somatic tissues to exogenous GA_3_ or paclobutrazol varies among species and those substances may differ in impact on SE.

### Biosynthesis of bioactive GA_3_ accompanies somatic embryogenesis induction in *Medicago truncatula* M9-10a line

Since previous experiments suggested that endogenous gibberellins are required for embryogenic callus growth and embryo production in *Medicago truncatula* testing this hypothesis was crucial for determining the contents of bioactive GAs during the SE progress in tissues initiated from embryogenic (M9-10a) and non-embryogenic (M9) explants’ leaves. Tissues of both lines contained GAs, precursors and active derivatives of the 13-hydroxylation and non-13-hydroxylation pathways (Figs 1 and 5, S1 Fig). However, only in embryogenic tissues, was the 13-hydroxylation pathway activated with a significant increase in GA_53_ and GA_19_ intermediates after the first week of induction. It was associated with an increase in only one bioactive gibberellin, GA_3_, observed after the second week of induction (Fig 5). Interestingly, changes in the gibberellin content coincided with a rapid growth of callus in the last two weeks of the induction phase (Fig 2b and 2c). Our findings showed, the entire bioactive gibberellin biosynthesis pathways during the induction phase in the embryogenic and non-embryogenic lines and specific activation of the 13-hydroxylation pathway resulting from an increase in bioactive GA_3_. It suggests that it is mainly GA_3_ that may participate in SE induction in *Medicago truncatula*. Jimenez and Bangerth [45] reported that the contents of GAs (GA_1_+GA_3_+GA_20_) in 7-week-old embryogenic callus of maize were significantly higher than in the non-embryogenic line, which partially agrees with our results. However, the purpose of gibberellin biosynthesis activation is not clear. Earlier studies demonstrated that, during the zygotic embryogenesis, endogenous gibberellins were required mainly at the early stages of embryo development, and may be related to the suspensor. The suspensor of embryos at the globular stage was found to contain several plant hormones such as gibberellins (GAs), auxin, cytokinin and abscisic acid [81]. It was also shown that GA_1_, GA_4_, GA_5_, GA_6_, GA_8_ and GA_44_ were present in suspensors of *Phaseolus coccineus* L., and that GAs production in the suspensor was important for appropriate development of the embryo [82–84]. Furthermore, in the suspensor of that plant was present mRNAs encoding GA3-oxidase, the key bioactive gibberellin biosynthesis enzyme [85]. GA_3_ and *GA3-oxidase* mRNA were also accumulated in suspensors of other legumes, e.g. *Glycine max* and *Cytisus laburnum* [reviewed 86], but GAs and mRNAs were not accumulated in the small *Arabidopsis* suspensor [87], which suggests that GA biosynthesis is not a common feature of all suspensors. This also suggests that an increase in selected gibberellins is a common feature during the early zygotic embryogenesis in the *Fabaceae*. Although it is not possible to distinguish a suspensor during the *Medicago* SE, an increase in the bioactive GA_3_ content seems to be crucial for induction of the process.

### Gibberellin biosynthesis in *Medicago truncatuala* is performed by enzymes coded by known orthologous genes

In addition to the great progress in sequencing of many plant genomes, achieved during the last decade, numerous genes still remain poorly annotated. Further examination of gene expression is required to perform alignments and construct phylogenetic trees for genes encoding enzymes responsible for the biosynthesis of precursors and bioactive gibberellins in *Medicago truncatula*. Orthologous protein sequences from *Arabidopsis thaliana* were used as well-defined models for gibberellin biosynthesis pathways. In *Arabidopsis*, the first three genes, *CPS*, *KS* and *KO*, which encode the enzymes that catalyze early steps of gibberellin biosynthesis in plastids are represented by single genes, and their inactivation by mutation resulted in dwarf phenotypes [7]. Within 20 *Medicago truncatula* gibberellin biosynthesis genes analyzed in this study (S1 Table), both *CPS* and *KO* were represented by a single gene each. However, the *Medicago truncatula* genome contains two hypothetical genes (*MtKS* and *MtKS-like*) for the *KS* gene, but only *MtKS* was transcriptionally active during induction phase. Following enzymes, KAOs, located in endoplasmic reticulum, catalyze reactions leading to GA12-aldehyde and GA12 and are coded by two genes in *Arabidopsis thaliana* [7]. Similar results were obtained for *Medicago truncatula*, which is consistent with data obtained for another *Fabaceae* member, *Pisum sativum*, where two *KAO* genes, too, were confirmed by Davidson *et al*. [88]. KAO proteins belong to cytochrome P450 monooxygenases (CYP88A) and have a high degree of similarity to β-amyrin 11-oxidases (CYP88D); however, only the latter subfamily was proposed to be specific to the *Fabaceae* [89]. Our results confirmed this finding. The databases contained neither *Arabidopsis thaliana*, *Brasica napus* nor *Oryza sativa* sequences. On the contrary, several genes were present in the *Fabaceae* genomes of *Medicago truncatula*, *Cicer arietinum*, *Glycine max* and *Phaseolus vulgaris*. Interestingly, three genes coding β-amyrin-oxidase in *Medicago truncatula* were transcribed during the SE progress. It has to be emphasized that not all the pathways are identical in the model plant *Arabidopsis thaliana* and other, not so closely related, plants. There are few literature data concerning the cytochrome CYP714 family and their role in conversion of GA_12_ via 13-oxidation to GA_53_. The *Arabidopsis* genome features two genes, *AtCYP714A1* and *AtCYP714A2*, which code enzymes of main functions in GA_12_ inactivation and steviol biosynthesis, respectively [11]. Only one of the identified *Medicago truncatula CYP714* genes (*MtCYP714A1*) segregated in the same subgroup with *Arabidopis* genes and single genes of other *Fabaceae* species analyzed. This might suggest that the enzymes function in *Medicago truncatula* in one of these processes, but further analyses are required. Interestingly, only one gene (*MtCYP714A2*) which segregated with the *Cicer* and *Glycine* genes does not have any counterpart in either *Arabidopsis thaliana* or *Oryza sativa*. Although it is not possible to propose any function for this gene, its expression was greatly stimulated as SE progressed. *CYP714B1* and *CYP714B2* genes have been recently identified in rice and confirmed to have GA13-oxidase activity of their protein products [10]. Both *MtCYP714C1* and *MtCYP714C2* segregated to the subgroup with these *Oryza* proteins; the sequence similarity may point to these genes as candidate genes for GA13-oxidases in *Medicago truncatula* nevertheless, further analyses should be performed. Genes of the *GA20ox* family encode enzymes active in cytosol and have four members in rice, five in *Arabidopsis*, seven in grapevine and eight in soybean [90–93]. In this work, eight *Medicago truncatula* genes coding gibberellin 20-oxidases were identified; similarly, nine genes in closely related *Cicer arietinum* were found. GA20-oxidases segregate into two separate subgroups specific for *Arabidopsis* and *Fabaceae*. Han and Zhu [92] suggested that this may be the effect of multiple functionalization of GA20oxidases, which can lead to sequence divergence and functional differentiation between plant species. Only four of *Medicago truncatula* genes were transcriptonally active during SE, which might support this statement. Two *Medicago truncatula* GA3-oxidase genes analyzed in this study and already deposited in GenBank are annotated as *Medicago truncatula* gibberellin 2-beta-dioxygenase, which suggests–according to the Enzyme Commission nomenclature number EC 1.14.11.13 –that these enzymes catalyze reaction in which bioactive gibberellins are converted to 2beta-hydroxygibberellines (gibberellin1 + 2-oxoglutarate + O_2_ ⇌ 2beta-hydroxygibberellin1 + succinate + CO_2_). However, our phylogenetical analysis confirmed both to be gibberellin 3-oxidases, i.e. enzymes responsible for biosynthesis of bioactive gibberellins, but not for their inactivation. One of the *MtGA3ox* genes was previously shown in *Medicago truncatula* to be a part of molecular response during the early stages of AM fungal interactions [94], and is identical with the *MtGA3ox2* gene from our study. To solve the problem of proper gene naming, Steele *et al*. [95] constructed the first extensive phylogenetic tree from *GA3ox* genes of eighty-seven *Medicago* species. The only *Medicago truncatula* gene included in that study was *MtGA3ox1* and this particular gene is identically annotated also in our study. *Arabidopsis thaliana* has four *GA3ox* genes, while *Oryza sativa* only two [7], which in the latter case resembles the number of these genes in *Medicago truncatula*. Interestingly, *Pisum sativum* and *Cicer arietinum*, the close *Medicago* counterparts, also have only two *GA3ox* genes in their genomes.

### Expression patterns of genes coding enzymes active in gibberellin biosynthesis reflect changes in endogenous gibberellin contents

To confirm the accumulation of GA_53_ (Fig 5), the main precursor of bioactive GAs biosynthesis in the 13-hydroxylation pathway (Fig 1), observed in the embryogenic M9-10a variant, expression patterns of appropriate genes were analyzed. Of all the genes tested, only *MtCPS* was differentially expressed in the two lines (Fig 6, S3 Fig). Expression of this gene in initial leaf explants was higher in the non-embryogenic line, but after two days of induction it drastically decreased in both lines, to finally, after the first week, increase in the embryogenic line only. CPS is regarded as a “gatekeeper” [14, 96] to ensure the flux of substrates for gibberellin biosynthesis; However, the process requires also KS [97]. *MtKS* was almost equally expressed in both lines, like *MtKO*, with the exception of *MtKAO* being up-regulated in the non-embryogenic line. These findings indicate that *MtCPS* activity might be partially responsible for GA_53_ accumulation during SE induction phase in the embryogenic line. Although LC-MS analysis of gibberellin metabolites has not established the presence of either ald-GA_12_ or GA_12_, it was very important to determine which product of the *Medicago truncatula CYP714* genes identified (S2d Fig) was responsible for biosynthesis of GA_53_, the product of GA_12_ 13-oxidation. This reaction is catalyzed by the family of cytochrome monooxygenases CYP714. So far, only one enzyme has been proposed in *Arabidopis* to have a minor GA13-ox activity and two enzymes in *Oryza sativa* with strong GA13-ox activity [10, 11]. Among four *CYP714* genes in *Medicago truncatula*, it is only the expression profile of *MtCYP714A1* and *MtCYP714C1* that coincides with the increase in GA_53_ after one week of induction in the embryogenic line (Figs 7 and 5). Interestingly, the first gene is similar to *Arabidopsis thaliana CYP714A1* and *CYP714A2* genes coding enzymes mainly responsible for catabolic activity. On the other hand, *MtCYP714C1* is similar to the *Oryza sativa CYP714* genes coding enzymes with GA13-oxidase activity. On the basis of transcription profiles it is not possible to determine which of the enzyme-coding *Medicago* genes may ensure further biosynthesis of GA_53_, particularly because expression of both genes is specifically induced at day 7 in the embryogenic and non-embryogenic line alike. The only difference in expression profiles observed between both lines concerns *CYP714A1*, when in the embryogenic line at day 14 and 21 expression was significantly higher, compared to the non-embryogenic line (Fig 7), which coincided with elevated amounts of GA_53_ at the same time-points (Fig 5). The most notable impact on biosynthesis of bioactive gibberellins is linked to spatio-temporal activity of 2-oxoglutarate-dependent dioxygenases (2ODDs) which include GA20oxidases and GA3oxidases. Expression profiles of *GA20ox* genes during *Arabidopsis* development revealed that three out of the 5 genes are highly expressed during vegetative and early reproductive development, which suggests functional redundancy of those genes [91]. The SE induction phase generally showed a higher transcriptional activities of three genes, *MtGA20ox1*, *MtGA20ox2*, *MtGA20ox7* and significantly lower activities of *MtGA20ox4* and *MtGA20ox6* in tissues of both lines (Fig 8), which might suggest that functional redundancy may also be the case in *Medicago truncatula*. Since gibberellin 20-oxidases catalyze multistep reaction in two GAs biosynthesis pathways leading from GA_12_ to GA_9_ and from GA_53_ to GA_20_, this makes it very hard to distinguish their catalytic specificity to particular substrates, and expression profiles may only suggest the most probable candidates for further analysis. Results from determinations of endogenous gibberellins showed a distinct, strong increase in the amount of GA_19_ metabolite between days 2 and 7 within the first week of SE induction only in the embryogenic line. This coincided with increased expression of the *MtGA20ox2* gene observed as early as on the second day, and mRNA was also the most abundant from all *GA20ox* genes tested during this period. However, the expression pattern of this gene is not restricted to the embryogenic line. Only two of *GA20ox* genes were differentially expressed in the embryogenic and non-embryogenic line, the first–*MtGA20ox1* –on day 7 and 14 showed 2-fold and 5-fold higher expression, respectively, in the non-embryogenic line, and the second–*MtGA20ox4* –with a 3-fold higher expression on day 14 in the embryogenic line (Fig 8). During SE in rice, *GA20ox1* was up-regulated exclusively in the embryogenic *japonica* subspecies [98]. Our results partially confirmed this finding. Genes of *GA20-oxidases* are activated during the induction phase of *Medicago truncatula* SE, but it was not possible to determine significant differences between the two lines. GA3-oxidases in plants catalyze the terminal biosynthetic steps ending with bioactive gibberellins including GA_4_, GA_7_, GA_1_, GA_3_ and increase in their contents should be at least partially reflected by an increase in *GA3ox* mRNA. Our results confirmed specific activation of *MtGA3ox1* after the first week of induction only in the embryogenic (M9-10a) line, and on subsequent days 7 and 14 expression in this line was 5-fold and 7-fold higher, respectively than in the non-embryogenic line (Fig 9). Interestingly, expression of the second gene *MtGA3ox2* was 10-fold higher in the M9-10a embryogenic line, but only on day 14 of the induction phase. Transcriptional activity of both genes coincides with an increased quantity of bioactive GA_3_, which is for the first time shown for plant SE. In carrot SE, expression of *DcGA3ox* genes was related only to induced embryogenic suspension cultures starting from the induction stage and lasting even to the globular stage of the differentiation phase [43]. Similar results were obtained for embryogenic *Oryza japonica*, while during the SE induction phase the *GA3ox2* gene was up-regulated specifically in this embryogenic subspecies [98]. Biosynthesis of bioactive gibberellins and their importance for SE is probably underestimated, but it should be considered as one of crucial factors. Support is provided by the most recent data from experiments with rice zygotic embryogenesis [99] whereby in the embryo-lethal rice mutant (*osmpk6*) impaired expression of *GA20ox* and *GA3ox* genes affected early stages of embryo development.

### Exogenous GA_3_ inhibits expression of *MtBBM* gene in embryogenic callus

BABY BOOM (BBM) was originally isolated from *Brassica* embryogenic microspore cultures; when ectopically expressed in *Arabidopsis*, induced spontaneous SE [100]. Although BBM was proven to be capable of inducing SE in numerous plant species [57, 101, 102], only few authors focus on BBM utility as a suitable marker to monitor SE progress [60, 103]. Our results confirmed transcriptional induction of *MtBBM* only in callus of the embryogenic M9-10a line, which is consistent with results from *Theobroma cacao* SE where expression of *TcBBM* was significantly higher in undifferentiated embryogenic callus, compared to non-embryogenic callus. *MtBBM* expression was highly time-dependent, and transcripts appeared exclusively in the embryogenic M9-10a variant after one week of induction. *BBM* expression was previously analyzed also in a highly embryogenic 2HA line of *Medicago truncatula*, but not for the purpose of examining the SE progress [61]. Although, despite the fact of differences in the induction medium composition and genotypes, *MtBBM* transcripts appeared also as early as one week after initiation of the induction phase. The appearance of *MtBBM* transcripts may be the first evidence of embryo formation, as suggested by data obtained by Horstman *et al*. [104]; in their study, *BBM* expression examined during early zygotic embryogenesis of *Arabidopsis thaliana* showed that transcripts were detectable starting from the 4-celled stage until the globular stage and then located in basal region of the heart stage embryo. It is reasonable to adopt (with minor modifications) a model of action during SE proposed by Rose *et al*. [78, 79] who divided the process into three distinct phases: dedifferentiation, expression and embryo development. Dedifferentiation occurs when the genetic program is changed (days 3–7), followed by the expression phase when the population of totipotent stem cells starts to proliferate, accompanied by expression of SE- specific genes (days 10–15), and finally development of embryos starts from asymmetrical cell division. We have previously shown [105] that during SE induction in *Medicago truncatula* on day 7 and 14, embryogenic line explants showed elevated transcriptional activity of *MtLEC1* and *MtL1L*. Probably also *MtBBM* expression reported in this work is not exceptional and may be related to the expression phase. A study on expression of the *BBM* gene during induction of *Coffea canephora* SE showed that application of 5-AzaC (inhibitor of DNA methyltransferase) to the induction medium resulted in decreased *BBM* expression and loss of SE [103]. This is a strong evidence for positive correlation between increased *BBM* expression and acquisition of callus tissue competence to SE. Very similarly, our results showed that when GA_3_ was added to the induction medium, *BBM* expression decreased and SE was impaired. Evidence supporting a negative effect of GA_3_ on gene expression of known SE markers was shown by Zheng *et al*. [56] in whose study, during soybean SE, exogenously applied gibberellin reduced expression of *AGL18*, *ABI3* and *FUS3*. This corresponds to our results, but we cannot determine the underlying mechanisms. The inhibitory effect of GA_3_ might be partially explained by the presence of elements regulated by gibberellin, which was confirmed in the promoter region of *Larix kaempferi BBM* gene [106], but further analyses of the *MtBBM* promoter region should be conducted along with chip-immunoassays experiments to validate this finding for *Medicago truncatula*. Nevertheless, it is worth to notice that paclobutrazol inhibited SE, but expression of *MtBBM* remained almost unchanged, which might suggest that, in addition to BBM activity, there are more components of the regulatory system that lead to acquisition of SE potential. This may probably be attributed to the fact that not only does paclobutrazol inhibit GAs biosynthesis, but it also inhibits the biosynthesis of ABA. Wang *et al*. [107] reported that PBZ reduced endogenous ABA levels in apple seedlings by about one-third. Nolan *et al*. [71] suggested that, in embryogenic *M*. *truncatula* 2HA line, a low exogenous ABA:GA ratio is required to be present during the SE induction phase; in different species, the expression of genes key for SE is probably related to differences in how the hormone networks optimize their expression.

## Conclusions

Our findings, elucidated entire bioactive gibberellin biosynthesis pathways during the induction phase in embryogenic and non-embryogenic lines of *Medicago truncatula*; specific activation of the 13-hydroxylation pathway resulted in an increase in bioactive GA_3_. We also fully annotated 20 *Medicago truncatula* orthologous genes coding enzymes which catalyze all the known steps of gibberellin biosynthesis. We showed that expression of only three genes, *MtCPS*, *MtGA3ox1* and *MtGA3ox2* was specific to embryogenic explants and reflected changes observed in GA_53_, GA_19_ and GA_3_ contents. Thus, all the results obtained suggest that of all the bioactive gibberellins present in tissues during the induction phase, only GA_3_ participates in SE initiation.

## Supporting information

S1 Fig Changes in gibberellin content. Changes in endogenous levels of non-13-hydroxy gibberellin metabolites during the induction phase in *Medicago truncatula* non-embryogenic genotype (M9) and variant with embryogenic phenotype (M9-10a). Two-way ANOVA with 0.05 confidence interval and Sidak post-hoc test; significance between groups indicated with * for P≤ 0.05, *** for P≤ 0.001 and **** for P≤ 0.0001. Bars indicate +/- SD (n = 3).
(PDF)

S2 Fig Phylogenetic relationships between amino acid sequences of gibberellin biosynthesis enzymes. Phylogenetic tree generated from protein sequences of (a)—CPS (ent-copalyl diphosphate synthase) and KS (ent-kaurene synthase), (b)—KO (ent-kaurene oxidase), (c)—KAO (ent- kaurenoic acid oxidase) and beta-amyrin 11-oxidase, (d)—CYP714 (Cytochrome 450 family, known also as Gibberellin 13-oxidases, GA13ox), (e)—GA20ox (gibberellin 20-oxidase) and GA3ox (gibberellin 3-oxidase), (f)—GA3ox (gibberellin 3-oxidase) representing phylogenetic relationships between plant taxa. Taxa terminologies are abbreviated using the first letter of the genus and first letter of the species name: At–*Arabidopsis thaliana*, Bn–*Brassica napus*, Ca–*Cicer arietinum*, Gm–*Glycine max*, Mt–*Medicago truncatula*, Os—*Oryza sativa*, Ps—*Pisum sativum*, Pv–*Phaseolus vulgaris*, Sl–*Solanum lycopersicum*. Phylogenetic analysis was performed using Geneious 6.1 software (Kearse et al. 2012) with Neighbor-Joining tree building method and Jukes-Cantor genetic distance model. Trees were resampled 1000 times using bootstrap method.
(PDF)

S3 Fig Relative gene expression of genes coding enzymes in early steps of gibberellin biosynthesis. Relative gene expression of *Medicago truncatula CPS (ent-copalyl diphosphate synthase*), *KS (ent-kaurene synthase)*, *KO (ent-kaurene oxidase)*, *KAO (ent-kaurenoic acid oxidase)* measured after first and second week of induction presented as a multiplication factor change in embryogenic variant (M9-10a) relative to non-embryogenic genotype (M9) set to 1. Statistical analyses were performed as two-tailed t-test with 0.05 confidence interval. Asterisks represent significance levels: *—P ≤ 0.05, **—P ≤ 0.01, ***—P ≤ 0.001 and ****—P ≤ 0.0001. Bars indicate +/- SD (n = 3).
(PDF)

S4 Fig Relative gene expression of genes coding enzymes in intermediate steps of gibberellin biosynthesis. Expression of *Medicago truncatula CYP714* genes (*Cytochrome 450 family*, known also as Gibberellin 13-oxidases, GA13ox) measured after first and second week of induction presented as a multiplication factor change in embryogenic variant (M9-10a) relative to non- embryogenic genotype (M9) set to 1. Statistical analysis were performed as two-tailed t-test with 0.05 confidence interval. Asterisks represent significance levels: *—P ≤ 0.05, ***—P ≤ 0.001, ****—P ≤ 0.0001 and ns for non-significant differences. Bars indicate +/- SD (n = 3).
(PDF)

S5 Fig Relative gene expression of genes coding *GA20oxidases* in late steps of gibberellin biosynthesis. Expression of *Medicago truncatula GA20ox (Gibberellin 20-oxidase)* genes measured after first and second week of induction presented as a multiplication factor change in embryogenic variant (M9-10a) relative to non-embryogenic genotype (M9) set to 1. Statistical analyses were performed as two-tailed t-test with 0.05 confidence interval. Asterisks represent significance levels: *—P ≤ 0.05, **—P ≤ 0.001 and ns for non-significant differences. Bars indicate +/- SD (n = 3).
(PDF)

S1 Table Real-time primers and conditions. Real-time primers and conditions. The table lists all the *Medicago truncatula* genes analyzed. The first and second column show protein name and abbreviated gene name. Subsequent columns show accession numbers in GeneBank for nucleotide and protein sequence and locus name according to *Medicago truncatula* IMGAG 4.0.1. The following two columns show the presence in the NGS transcriptome library and whether a qPCR product was detectable at any of the collection time-points tested. The last three columns refer to technical information on qPCR primers, e.g. sequence, length, melting temperature and product length. Formula used in primer express is nearest neighbor algorithm for Tm calculations algorithm Tm, expressed in Co, is calculated using the nearest-neighbor algorithm.
(XLSX)
