# Supplementary material for: Gene expression and metabolite profiling of gibberellin biosynthesis during induction of somatic embryogenesis in Medicago truncatula Gaertn
Source: PLoS One. 2017 Jul 27;12(7):e0182055. doi: 10.1371/journal.pone.0182055 (PMC5531487; doi:10.1371/journal.pone.0182055)
Supplement: S1 Fig — Changes in endogenous levels of non-13-hydroxy gibberellin metabolites during the induction phase in Medicago truncatula non-embryogenic genotype (M9) and variant with embryogenic phenotype (M9-10a). Two-way ANOVA with 0.05 confidence interval and Sidak post-hoc test; significance between groups indicated with * for P≤ 0.05, *** for P≤ 0.001 and **** for P≤ 0.0001. Bars indicate +/- SD (n = 3). (PDF) [file pone.0182055.s001.pdf]

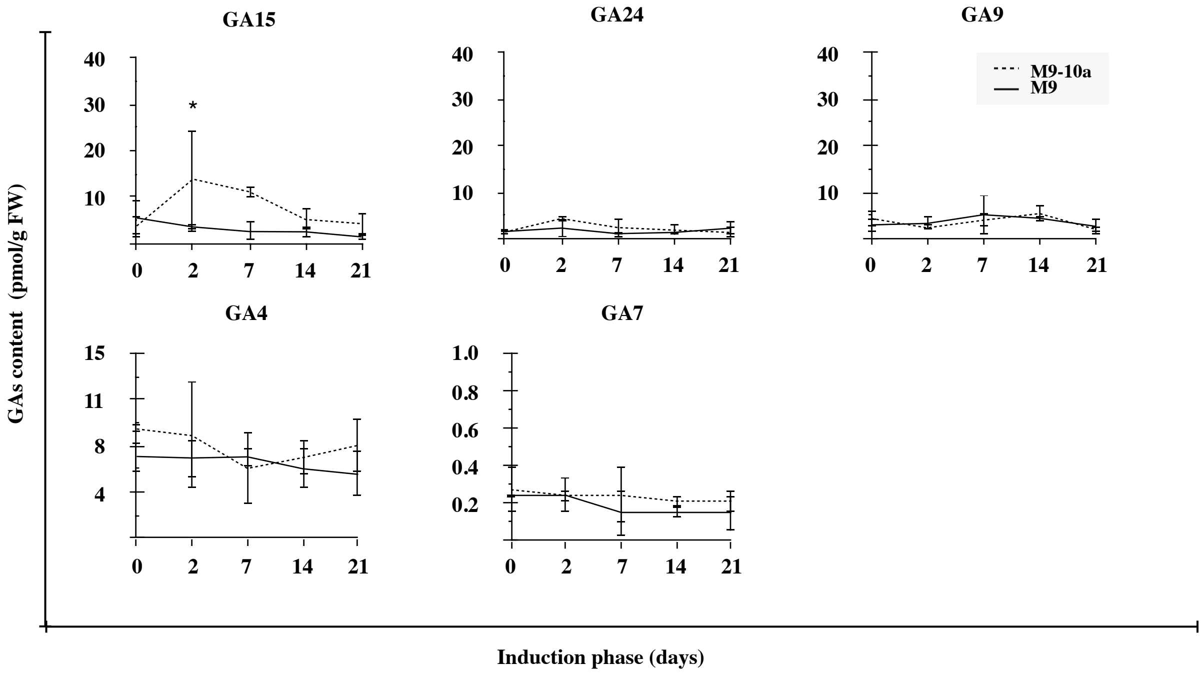

S1 Fig.

Changes in gibberellin content. Changes in endogenous levels of non-13-hydroxy gibberellin metabolites during the induction phase in *Medicago truncatula* non-embryogenic genotype (M9) and variant with embryogenic phenotype (M9-10a). Two-way ANOVA with 0.05 confidence interval and Sidak post-hoc test; significance between groups indicated with * for P≤ 0.05, *** for P≤ 0.001 and **** for P≤ 0.0001. Bars indicate +/- SD (n = 3).
