# Supplementary material for: Gene expression and metabolite profiling of gibberellin biosynthesis during induction of somatic embryogenesis in Medicago truncatula Gaertn
Source: PLoS One. 2017 Jul 27;12(7):e0182055. doi: 10.1371/journal.pone.0182055 (PMC5531487; doi:10.1371/journal.pone.0182055)
Supplement: S2 Fig — Phylogenetic tree generated from protein sequences of (a)—CPS (ent-copalyl diphosphate synthase) and KS (ent-kaurene synthase), (b)—KO (ent-kaurene oxidase), (c)—KAO (ent- kaurenoic acid oxidase) and beta-amyrin 11-oxidase, (d)—CYP714 (Cytochrome 450 family, known also as Gibberellin 13-oxidases, GA13ox), (e)—GA20ox (gibberellin 20-oxidase) and GA3ox (gibberellin 3-oxidase), (f)—GA3ox (gibberellin 3-oxidase) representing phylogenetic relationships between plant taxa. Taxa terminologies are abbreviated using the first letter of the genus and first letter of the species name: At–Arabidopsis thaliana, Bn–Brassica napus, Ca–Cicer arietinum, Gm–Glycine max, Mt–Medicago truncatula, Os—Oryza sativa, Ps—Pisum sativum, Pv–Phaseolus vulgaris, Sl–Solanum lycopersicum. Phylogenetic analysis was performed using Geneious 6.1 software (Kearse et al. 2012) with Neighbor-Joining tree building method and Jukes-Cantor genetic distance model. Trees were resampled 1000 times using bootstrap method. (PDF) [file pone.0182055.s002.pdf]

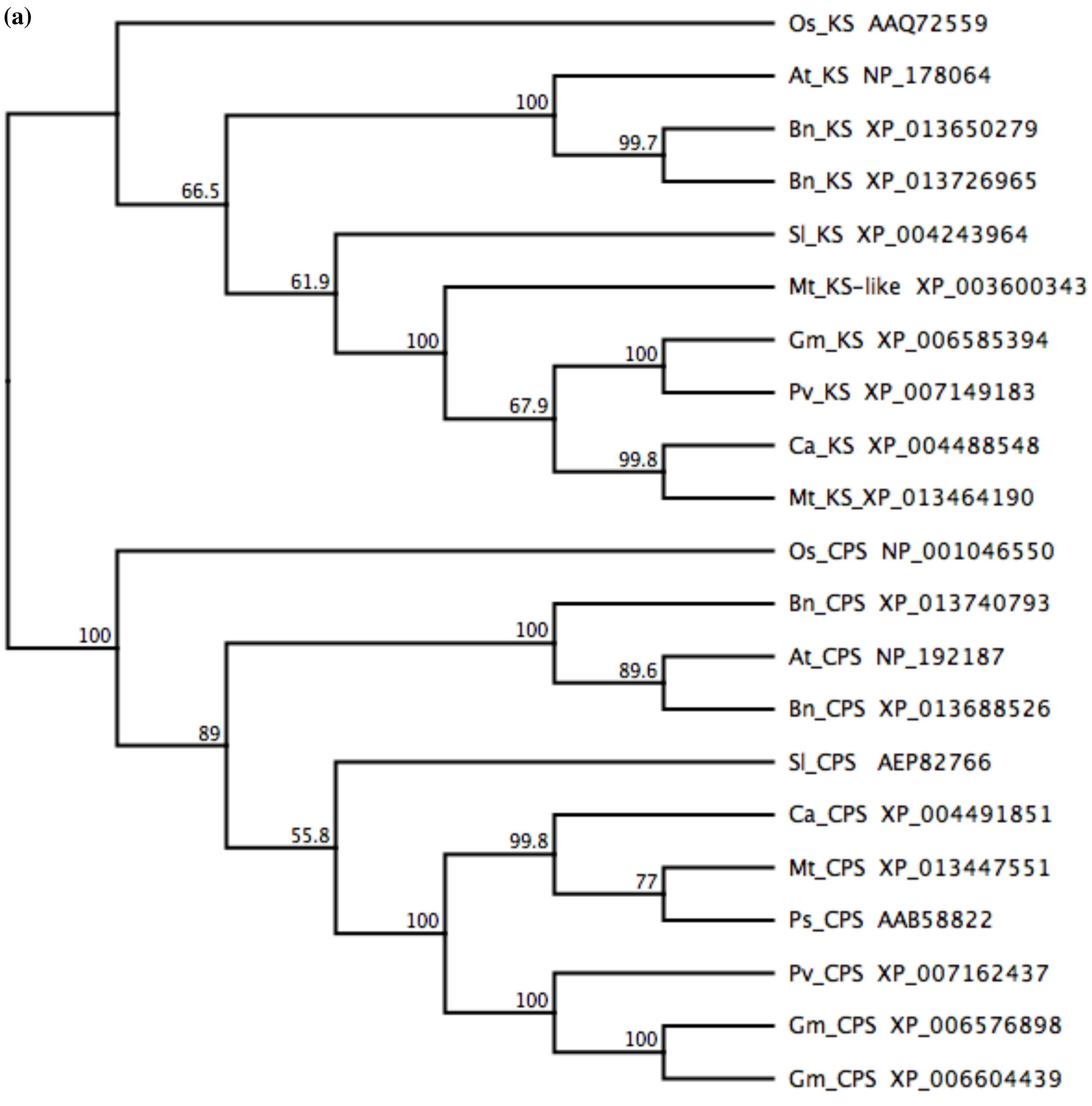

S2 Fig.

Phylogenetic relationships between amino acid sequences of gibberellin biosynthesis enzymes. Phylogenetic tree generated from protein sequences of (a) - CPS (ent-copalyl diphosphate synthase) and KS (ent-kaurene synthase), (b) - KO (ent-kaurene oxidase), (c) - KAO (ent-kaurenoic acid oxidase) and beta-amyrin 11-oxidase*,* (d) - CYP714 (Cytochrome 450 family, known also as Gibberellin 13-oxidases, GA13ox), (e) - GA20ox (gibberellin 20-oxidase) and GA3ox (gibberellin 3-oxidase), (f) - GA3ox (gibberellin 3-oxidase) representing phylogenetic relationships between plant taxa. Taxa terminologies are abbreviated using the first letter of the genus and first letter of the species name: At – *Arabidopsis thaliana*, Bn – *Brassica napus*, Ca – *Cicer arietinum,* Gm – *Glycine max,* Mt – *Medicago truncatula,* Os - *Oryza sativa*, Ps - *Pisum sativum*, Pv – *Phaseolus vulgaris,* Sl – *Solanum lycopersicum.* Phylogenetic analysis was performed using Geneious 6.1 software with Neighbor-Joining tree building method and Jukes-Cantor genetic distance model. Trees were resampled 1000 times using bootstrap method.

(b)

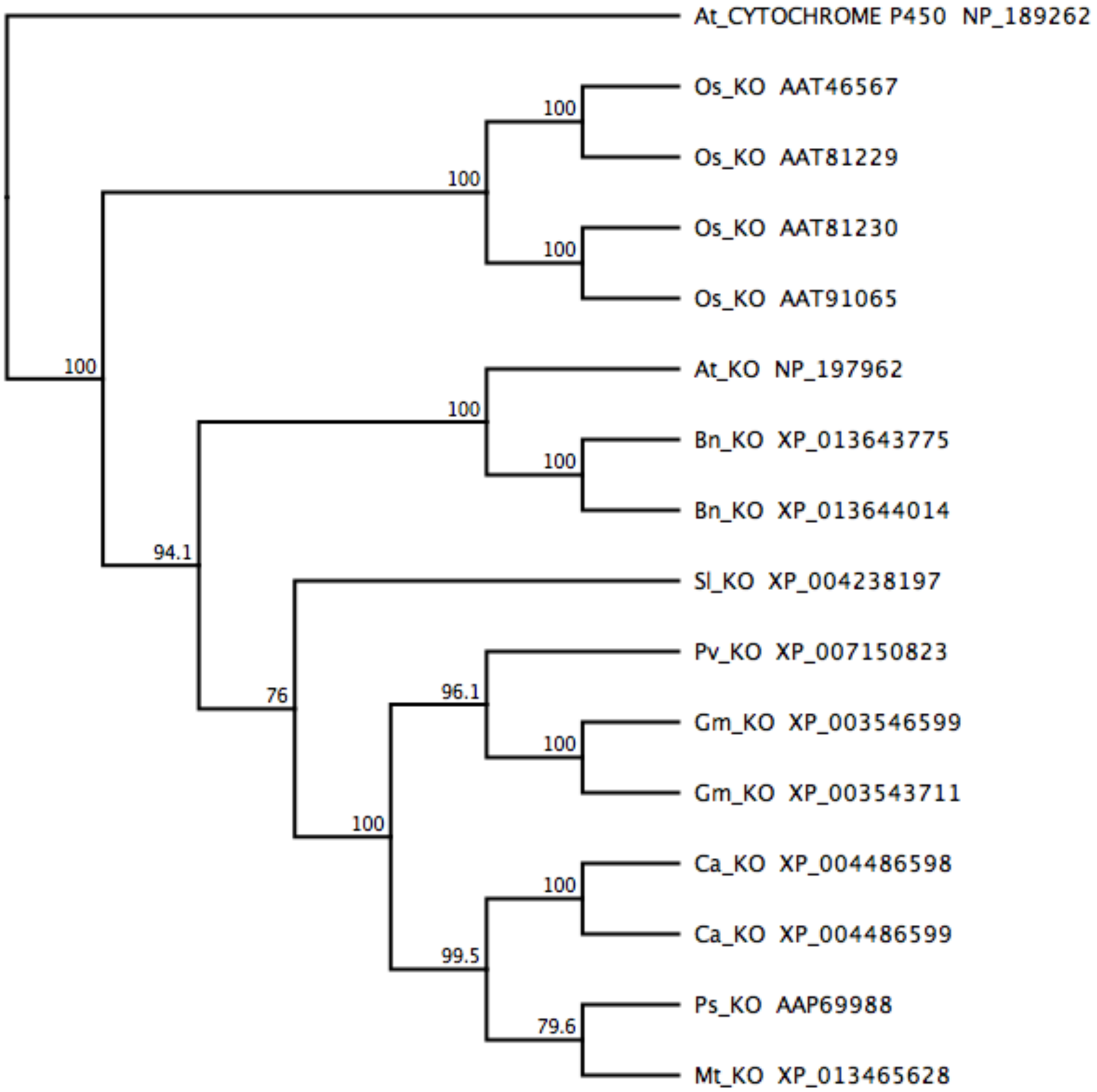
At_CYTOCHROME P450 NP_189262
Os_KO AAT46567
Os_KO AAT81229
Os_KO AAT81230
Os_KO AAT91065
At_KO NP_197962
Bn_KO XP_013643775
Bn_KO XP_013644014
Sl_KO XP_004238197
Pv_KO XP_007150823
Gm_KO XP_003546599
Gm_KO XP_003543711
Ca_KO XP_004486598
Ca_KO XP_004486599
Ps_KO AAP69988
Mt_KO XP_013465628
100
100
100
100
100
100
94.1
76
96.1
100
100
100
99.5
79.6

(c)

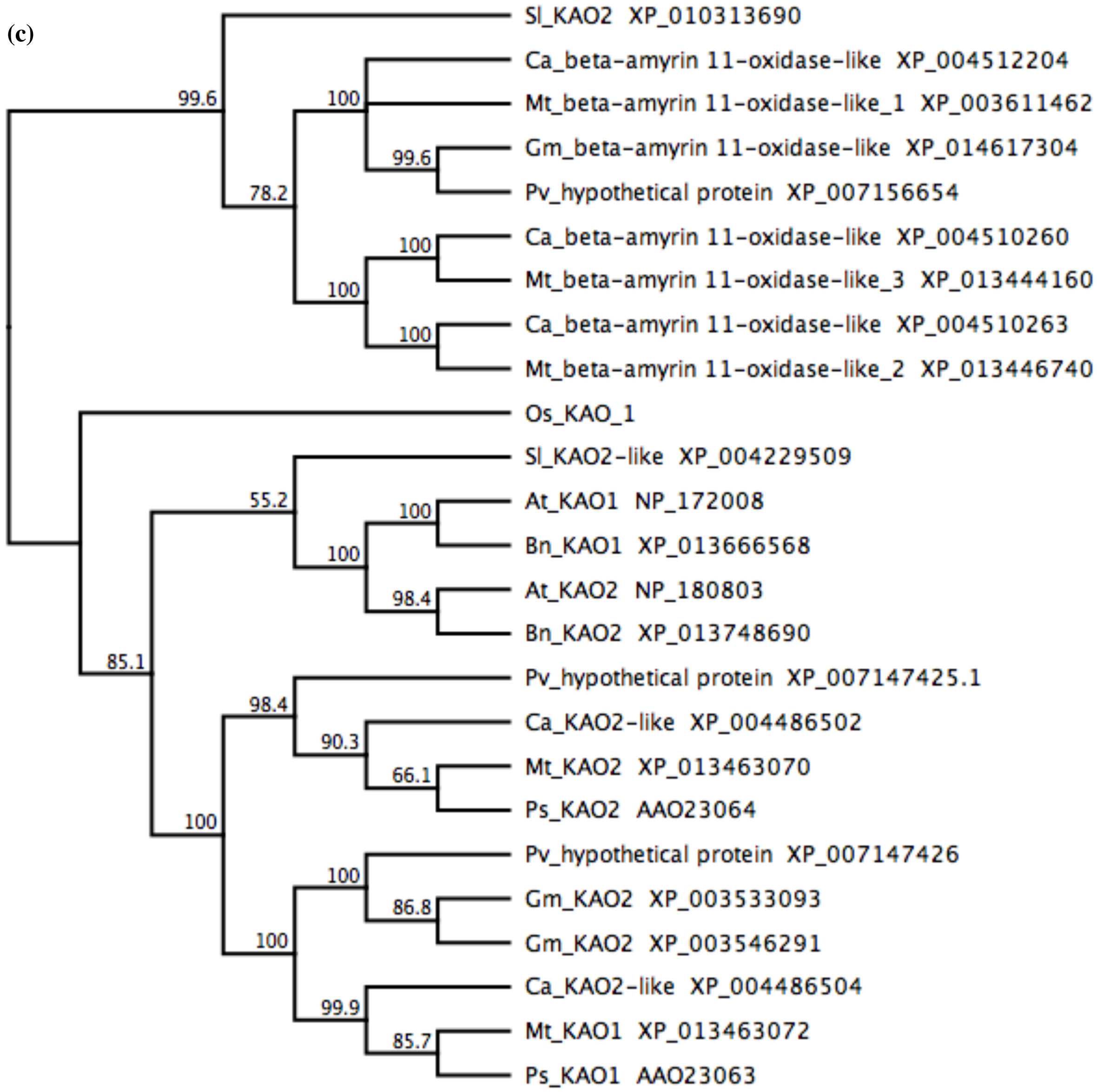

Sl_KAO2 XP_010313690
Ca_beta-amyrin 11-oxidase-like XP_004512204
Mt_beta-amyrin 11-oxidase-like_1 XP_003611462
Gm_beta-amyrin 11-oxidase-like XP_014617304
Pv_hypothetical protein XP_007156654
Ca_beta-amyrin 11-oxidase-like XP_004510260
Mt_beta-amyrin 11-oxidase-like_3 XP_013444160
Ca_beta-amyrin 11-oxidase-like XP_004510263
Mt_beta-amyrin 11-oxidase-like_2 XP_013446740
Os_KAO_1
Sl_KAO2-like XP_004229509
At_KAO1 NP_172008
Bn_KAO1 XP_013666568
At_KAO2 NP_180803
Bn_KAO2 XP_013748690
Pv_hypothetical protein XP_007147425.1
Ca_KAO2-like XP_004486502
Mt_KAO2 XP_013463070
Ps_KAO2 AAO23064
Pv_hypothetical protein XP_007147426
Gm_KAO2 XP_003533093
Gm_KAO2 XP_003546291
Ca_KAO2-like XP_004486504
Mt_KAO1 XP_013463072
Ps_KAO1 AAO23063
99.6
100
99.6
78.2
100
100
100
55.2
100
100
98.4
85.1
98.4
90.3
66.1
100
100
86.8
100
99.9
85.7

(d)

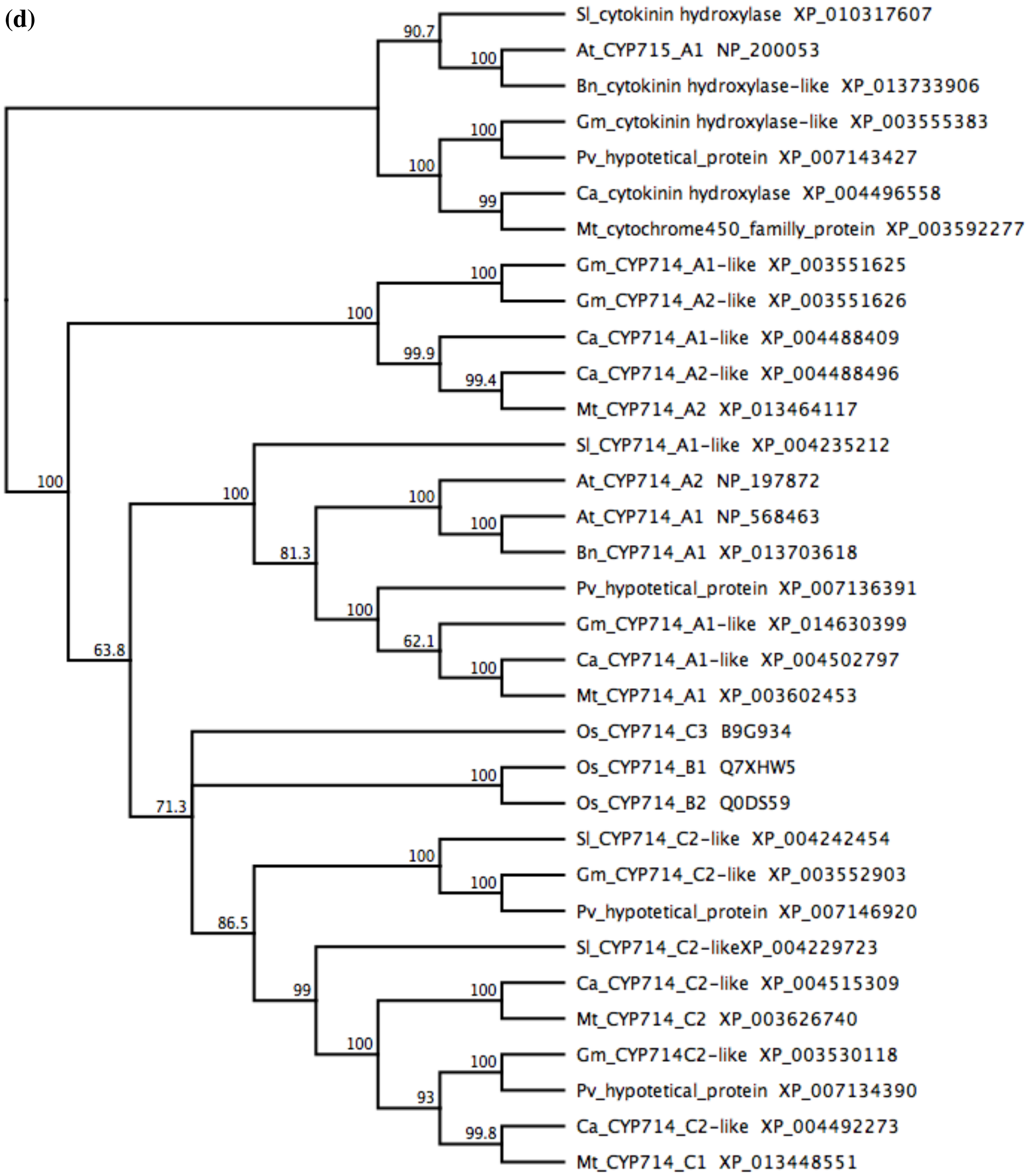
Sl_cytokinin hydroxylase XP_010317607
At_CYP715_A1 NP_200053
Bn_cytokinin hydroxylase-like XP_013733906
Gm_cytokinin hydroxylase-like XP_003555383
Pv_hypotetical_protein XP_007143427
Ca_cytokinin hydroxylase XP_004496558
Mt_cytochrome450_familly_protein XP_003592277
Gm_CYP714_A1-like XP_003551625
Gm_CYP714_A2-like XP_003551626
Ca_CYP714_A1-like XP_004488409
Ca_CYP714_A2-like XP_004488496
Mt_CYP714_A2 XP_013464117
Sl_CYP714_A1-like XP_004235212
At_CYP714_A2 NP_197872
At_CYP714_A1 NP_568463
Bn_CYP714_A1 XP_013703618
Pv_hypotetical_protein XP_007136391
Gm_CYP714_A1-like XP_014630399
Ca_CYP714_A1-like XP_004502797
Mt_CYP714_A1 XP_003602453
Os_CYP714_C3 B9G934
Os_CYP714_B1 Q7XHW5
Os_CYP714_B2 Q0DS59
Sl_CYP714_C2-like XP_004242454
Gm_CYP714_C2-like XP_003552903
Pv_hypotetical_protein XP_007146920
Sl_CYP714_C2-likeXP_004229723
Ca_CYP714_C2-like XP_004515309
Mt_CYP714_C2 XP_003626740
Gm_CYP714C2-like XP_003530118
Pv_hypotetical_protein XP_007134390
Ca_CYP714_C2-like XP_004492273
Mt_CYP714_C1 XP_013448551
90.7
100
100
100
99
100
100
99.9
99.4
100
100
100
100
81.3
100
62.1
100
63.8
100
71.3
100
100
86.5
99
100
100
100
93
99.8

(e)

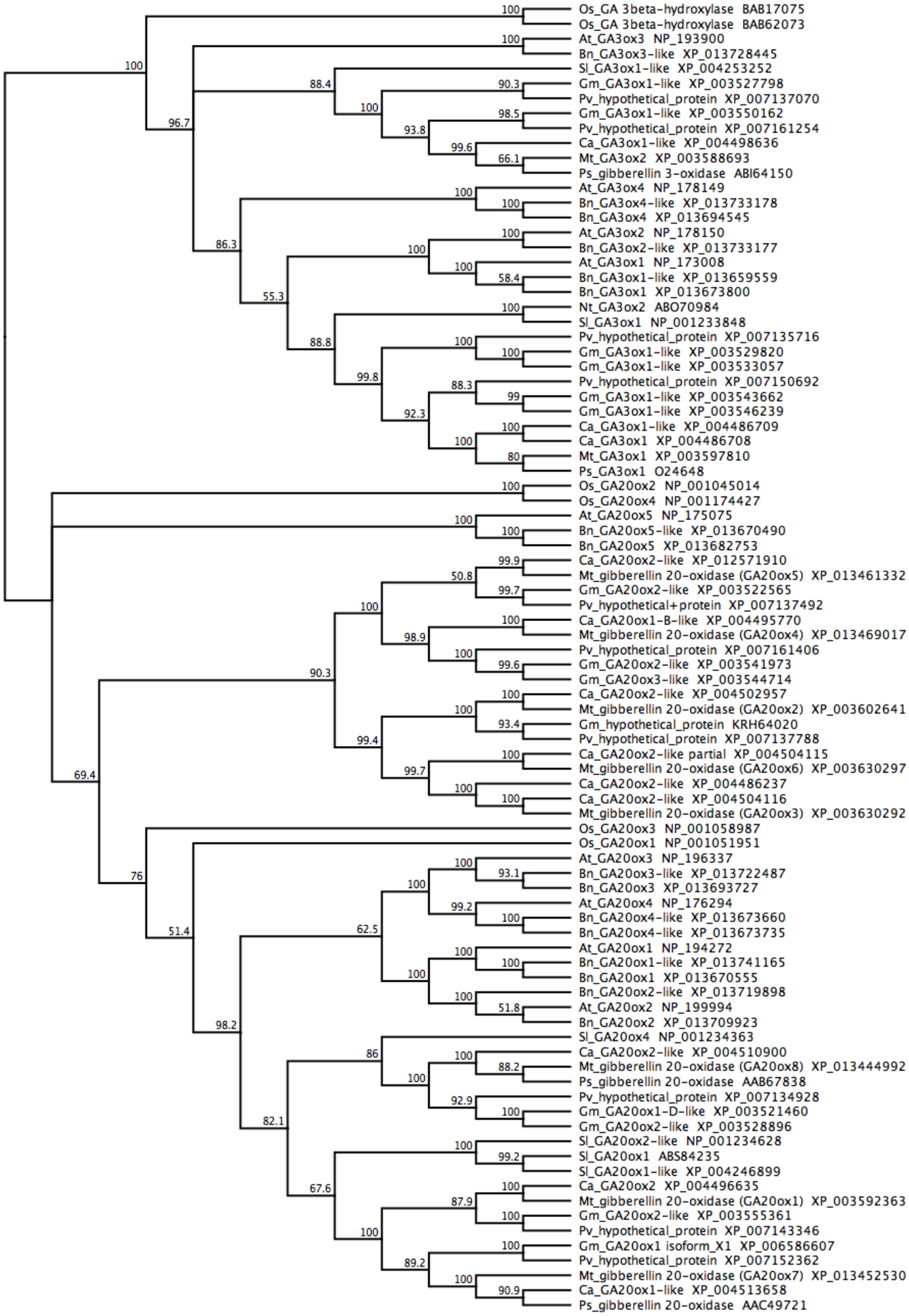
Os_GA 3beta-hydroxylase BAB17075
Os_GA 3beta-hydroxylase BAB62073
At_GA3ox3 NP_193900
Bn_GA3ox3-like XP_013728445
Sl_GA3ox1-like XP_004253252
Gm_GA3ox1-like XP_003527798
Pv_hypothetical_protein XP_007137070
Gm_GA3ox1-like XP_003550162
Pv_hypothetical_protein XP_007161254
Ca_GA3ox1-like XP_004498636
Mt_GA3ox2 XP_003588693
Ps_gibberellin 3-oxidase ABI64150
At_GA3ox4 NP_178149
Bn_GA3ox4-like XP_013733178
Bn_GA3ox4 XP_013694545
At_GA3ox2 NP_178150
Bn_GA3ox2-like XP_013733177
At_GA3ox1 NP_173008
Bn_GA3ox1-like XP_013659559
Bn_GA3ox1 XP_013673800
Nt_GA3ox2 ABO70984
Sl_GA3ox1 NP_001233848
Pv_hypothetical_protein XP_007135716
Gm_GA3ox1-like XP_003529820
Gm_GA3ox1-like XP_003533057
Pv_hypothetical_protein XP_007150692
Gm_GA3ox1-like XP_003543662
Gm_GA3ox1-like XP_003546239
Ca_GA3ox1-like XP_004486709
Ca_GA3ox1 XP_004486708
Mt_GA3ox1 XP_003597810
Ps_GA3ox1 O24648
Os_GA20ox2 NP_001045014
Os_GA20ox4 NP_001174427
At_GA20ox5 NP_175075
Bn_GA20ox5-like XP_013670490
Bn_GA20ox5 XP_013682753
Ca_GA20ox2-like XP_012571910
Mt_gibberellin 20-oxidase (GA20ox5) XP_013461332
Gm_GA20ox2-like XP_003522565
Pv_hypothetical+protein XP_007137492
Ca_GA20ox1-B-like XP_004495770
Mt_gibberellin 20-oxidase (GA20ox4) XP_013469017
Pv_hypothetical_protein XP_007161406
Gm_GA20ox2-like XP_003541973
Gm_GA20ox3-like XP_003544714
Ca_GA20ox2-like XP_004502957
Mt_gibberellin 20-oxidase (GA20ox2) XP_003602641
Gm_hypothetical_protein KRH64020
Pv_hypothetical_protein XP_007137788
Ca_GA20ox2-like partial XP_004504115
Mt_gibberellin 20-oxidase (GA20ox6) XP_003630297
Ca_GA20ox2-like XP_004486237
Ca_GA20ox2-like XP_004504116
Mt_gibberellin 20-oxidase (GA20ox3) XP_003630292
Os_GA20ox3 NP_001058987
Os_GA20ox1 NP_001051951
At_GA20ox3 NP_196337
Bn_GA20ox3-like XP_013722487
Bn_GA20ox3 XP_013693727
At_GA20ox4 NP_176294
Bn_GA20ox4-like XP_013673660
Bn_GA20ox4-like XP_013673735
At_GA20ox1 NP_194272
Bn_GA20ox1-like XP_013741165
Bn_GA20ox1 XP_013670555
Bn_GA20ox2-like XP_013719898
At_GA20ox2 NP_199994
Bn_GA20ox2 XP_013709923
Sl_GA20ox4 NP_001234363
Ca_GA20ox2-like XP_004510900
Mt_gibberellin 20-oxidase (GA20ox8) XP_013444992
Ps_gibberellin 20-oxidase AAB67838
Pv_hypothetical_protein XP_007134928
Gm_GA20ox1-D-like XP_003521460
Gm_GA20ox2-like XP_003528896
Sl_GA20ox2-like NP_001234628
Sl_GA20ox1 ABS84235
Sl_GA20ox1-like XP_004246899
Ca_GA20ox2 XP_004496635
Mt_gibberellin 20-oxidase (GA20ox1) XP_003592363
Gm_GA20ox2-like XP_003555361
Pv_hypothetical_protein XP_007143346
Gm_GA20ox1 isoform_X1 XP_006586607
Pv_hypothetical_protein XP_007152362
Mt_gibberellin 20-oxidase (GA20ox7) XP_013452530
Ca_GA20ox1-like XP_004513658
Ps_gibberellin 20-oxidase AAC49721

(f)

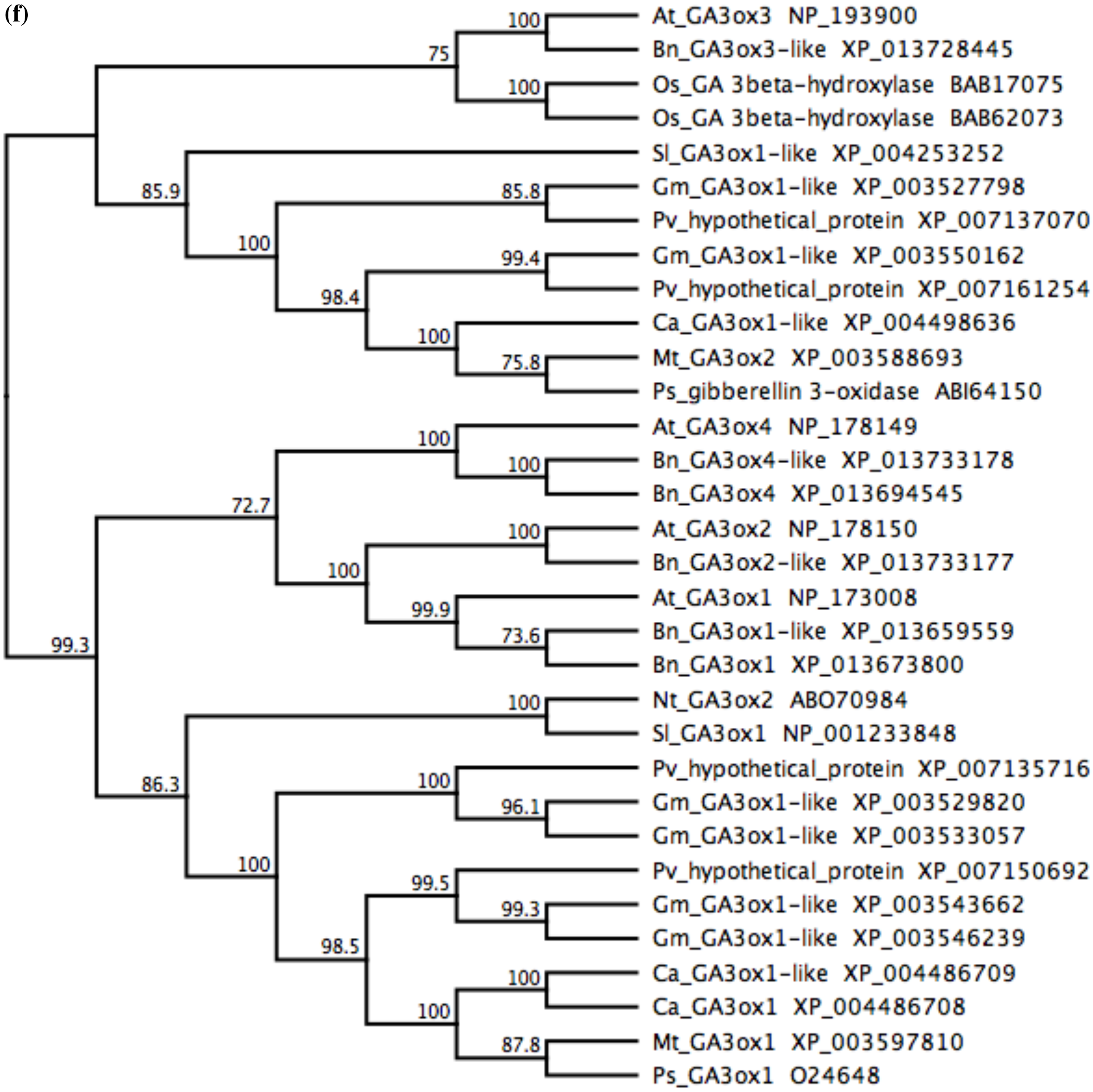
At_GA3ox3 NP_193900
Bn_GA3ox3-like XP_013728445
Os_GA 3beta-hydroxylase BAB17075
Os_GA 3beta-hydroxylase BAB62073
Sl_GA3ox1-like XP_004253252
Gm_GA3ox1-like XP_003527798
Pv_hypothetical_protein XP_007137070
Gm_GA3ox1-like XP_003550162
Pv_hypothetical_protein XP_007161254
Ca_GA3ox1-like XP_004498636
Mt_GA3ox2 XP_003588693
Ps_gibberellin 3-oxidase ABI64150
At_GA3ox4 NP_178149
Bn_GA3ox4-like XP_013733178
Bn_GA3ox4 XP_013694545
At_GA3ox2 NP_178150
Bn_GA3ox2-like XP_013733177
At_GA3ox1 NP_173008
Bn_GA3ox1-like XP_013659559
Bn_GA3ox1 XP_013673800
Nt_GA3ox2 ABO70984
Sl_GA3ox1 NP_001233848
Pv_hypothetical_protein XP_007135716
Gm_GA3ox1-like XP_003529820
Gm_GA3ox1-like XP_003533057
Pv_hypothetical_protein XP_007150692
Gm_GA3ox1-like XP_003543662
Gm_GA3ox1-like XP_003546239
Ca_GA3ox1-like XP_004486709
Ca_GA3ox1 XP_004486708
Mt_GA3ox1 XP_003597810
Ps_GA3ox1 O24648
100
75
100
85.9
85.8
100
99.4
98.4
100
75.8
100
100
72.7
100
100
99.9
73.6
99.3
100
86.3
100
96.1
100
99.5
99.3
98.5
100
100
87.8
