# Supplementary material for: Gene expression and metabolite profiling of gibberellin biosynthesis during induction of somatic embryogenesis in Medicago truncatula Gaertn
Source: PLoS One. 2017 Jul 27;12(7):e0182055. doi: 10.1371/journal.pone.0182055 (PMC5531487; doi:10.1371/journal.pone.0182055)
Supplement: S3 Fig — Relative gene expression of Medicago truncatula CPS (ent-copalyl diphosphate synthase), KS (ent-kaurene synthase), KO (ent-kaurene oxidase), KAO (ent-kaurenoic acid oxidase) measured after first and second week of induction presented as a multiplication factor change in embryogenic variant (M9-10a) relative to non-embryogenic genotype (M9) set to 1. Statistical analyses were performed as two-tailed t-test with 0.05 confidence interval. Asterisks represent significance levels: *—P ≤ 0.05, **—P ≤ 0.01, ***—P ≤ 0.001 and ****—P ≤ 0.0001. Bars indicate +/- SD (n = 3). (PDF) [file pone.0182055.s003.pdf]

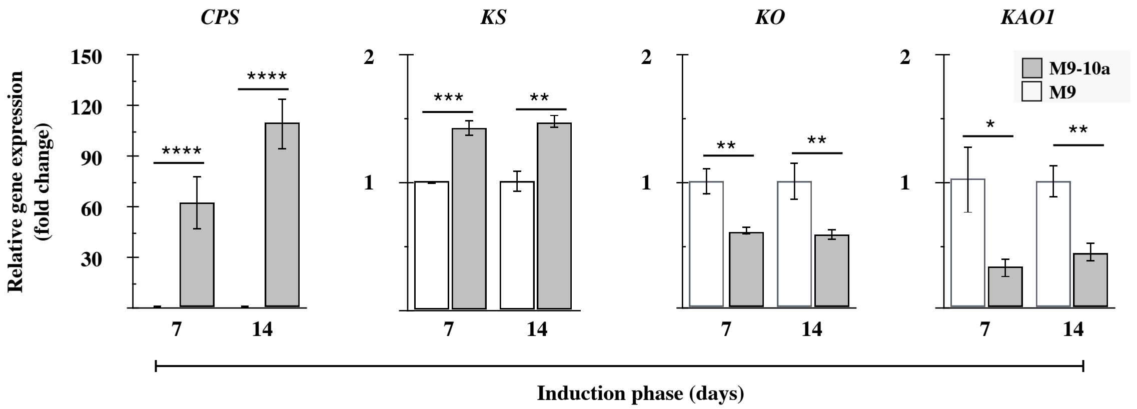

S3 Fig.

Relative gene expression of genes coding enzymes in early steps of gibberellin biosynthesis. Relative gene expression of *Medicago truncatula CPS (ent-copalyl diphosphate synthase*), *KS (ent-kaurene synthase), KO (ent-kaurene oxidase), KAO (ent-kaurenoic acid oxidase)* measured after first and second week of induction presented as a multiplication factor change in embryogenic variant (M9-10a) relative to non-embryogenic genotype (M9) set to 1. Statistical analyses were performed as two-tailed t-test with 0.05 confidence interval. Asterisks represent significance levels: * - $P \leq 0.05$, ** - $P \leq 0.01$, *** - $P \leq 0.001$ and **** - $P \leq 0.0001$. Bars indicate +/- SD (n = 3).
