# Supplementary material for: Gene expression and metabolite profiling of gibberellin biosynthesis during induction of somatic embryogenesis in Medicago truncatula Gaertn
Source: PLoS One. 2017 Jul 27;12(7):e0182055. doi: 10.1371/journal.pone.0182055 (PMC5531487; doi:10.1371/journal.pone.0182055)
Supplement: S4 Fig — Expression of Medicago truncatula CYP714 genes (Cytochrome 450 family, known also as Gibberellin 13-oxidases, GA13ox) measured after first and second week of induction presented as a multiplication factor change in embryogenic variant (M9-10a) relative to non- embryogenic genotype (M9) set to 1. Statistical analysis were performed as two-tailed t-test with 0.05 confidence interval. Asterisks represent significance levels: *—P ≤ 0.05, ***—P ≤ 0.001, ****—P ≤ 0.0001 and ns for non-significant differences. Bars indicate +/- SD (n = 3). (PDF) [file pone.0182055.s004.pdf]

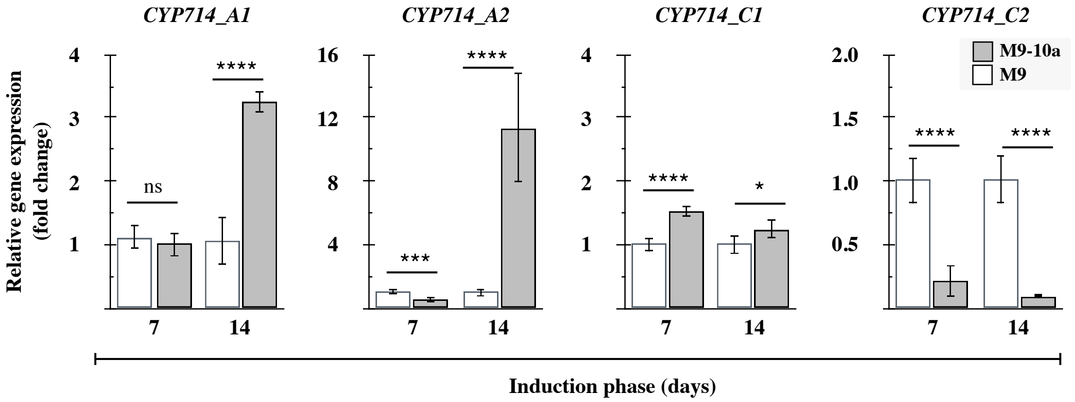

S4 Fig.

Relative gene expression of genes coding enzymes in intermediate steps of gibberellin biosynthesis. Expression of *Medicago truncatula CYP714* genes (*Cytochrome 450 family,* known also as Gibberellin 13-oxidases, GA13ox) measured after first and second week of induction presented as a multiplication factor change in embryogenic variant (M9-10a) relative to non-embryogenic genotype (M9) set to 1. Statistical analysis were performed as two-tailed t-test with 0.05 confidence interval. Asterisks represent significance levels: * - $P \leq 0.05$, *** - $P \leq 0.001$, **** - $P \leq 0.0001$ and ns for non-significant differences. Bars indicate +/- SD (n = 3).
